# Suppression of pyrrolidine ring biosynthesis and its effects on gene expression and subsequent accumulation of anatabine in leaves of tobacco (*N. tabacum* L.)

**DOI:** 10.1186/s12864-023-09588-8

**Published:** 2023-09-04

**Authors:** Kacper Piotr Kaminski, Lucien Bovet, Aurore Hilfiker, Helene Laparra, Joanne Schwaar, Nicolas Sierro, Gerhard Lang, Damien De Palo, Philippe Alexandre Guy, Csaba Laszlo, Simon Goepfert, Nikolai V. Ivanov

**Affiliations:** Philip Morris International R&D, Philip Morris Products S.A, Quai Jeanrenaud 5, CH-2000 Neuchâtel, Switzerland

**Keywords:** Anatabine, Nicotine, *Nicotiana tabacum*, MPO, PMT, A622, BBLs, Topping

## Abstract

**Background:**

Anatabine, although being one of four major tobacco alkaloids, is never accumulated in high quantity in any of the naturally occurring species from the *Nicotiana* genus. Previous studies therefore focused on transgenic approaches to synthetize anatabine, most notably by generating transgenic lines with suppressed putrescine methyltransferase (PMT) activity. This led to promising results, but the global gene expression of plants with such distinct metabolism has not been analyzed. In the current study, we describe how these plants respond to topping and the downstream effects on alkaloid biosynthesis.

**Results:**

The surge in anatabine accumulation in PMT transgenic lines after topping treatment and its effects on gene expression changes were analyzed. The results revealed increases in expression of isoflavone reductase-like (A622) and berberine bridge-like enzymes (BBLs) oxidoreductase genes, previously shown to be crucial for the final steps of nicotine biosynthesis. We also observed significantly higher methylputrescine oxidase (MPO) expression in all plants subjected to topping treatment. In order to investigate if MPO suppression would have the same effects as that of PMT, we generated transgenic plants. These plants with suppressed MPO expression showed an almost complete drop in leaf nicotine content, whereas leaf anatabine was observed to increase by a factor of ~ 1.6X.

**Conclusion:**

Our results are the first concrete evidence that suppression of MPO leads to decreased nicotine in favor of anatabine in tobacco roots and that this anatabine is successfully transported to tobacco leaves. Alkaloid transport in plants remains to be investigated to higher detail due to high variation of its efficiency among Nicotiana species and varieties of tobacco. Our research adds important step to better understand pyrrolidine ring biosynthesis and its effects on gene expression and subsequent accumulation of anatabine.

**Supplementary Information:**

The online version contains supplementary material available at 10.1186/s12864-023-09588-8.

## Background

Among various species of the *Nicotiana* genus, there are four major secondary metabolites (nicotine, nornicotine, anabasine, and anatabine) that constitute the vast majority of the total alkaloid pool [1, 2]. Nicotine is the major alkaloid for many of these species, including common tobacco (*N. tabacum* L.), which typically accumulates 2–4% of alkaloids in total dry weight. Nicotine accounts for approximately 90% of the alkaloid content, and nornicotine and anatabine comprise most of the remaining 10%. Despite extensive *Nicotiana* alkaloid pathway research, it is still unclear if anatabine biosynthesis is completely dependent on nicotine biosynthesis or requires dedicated enzymatic steps [3].

**Fig. 1:**
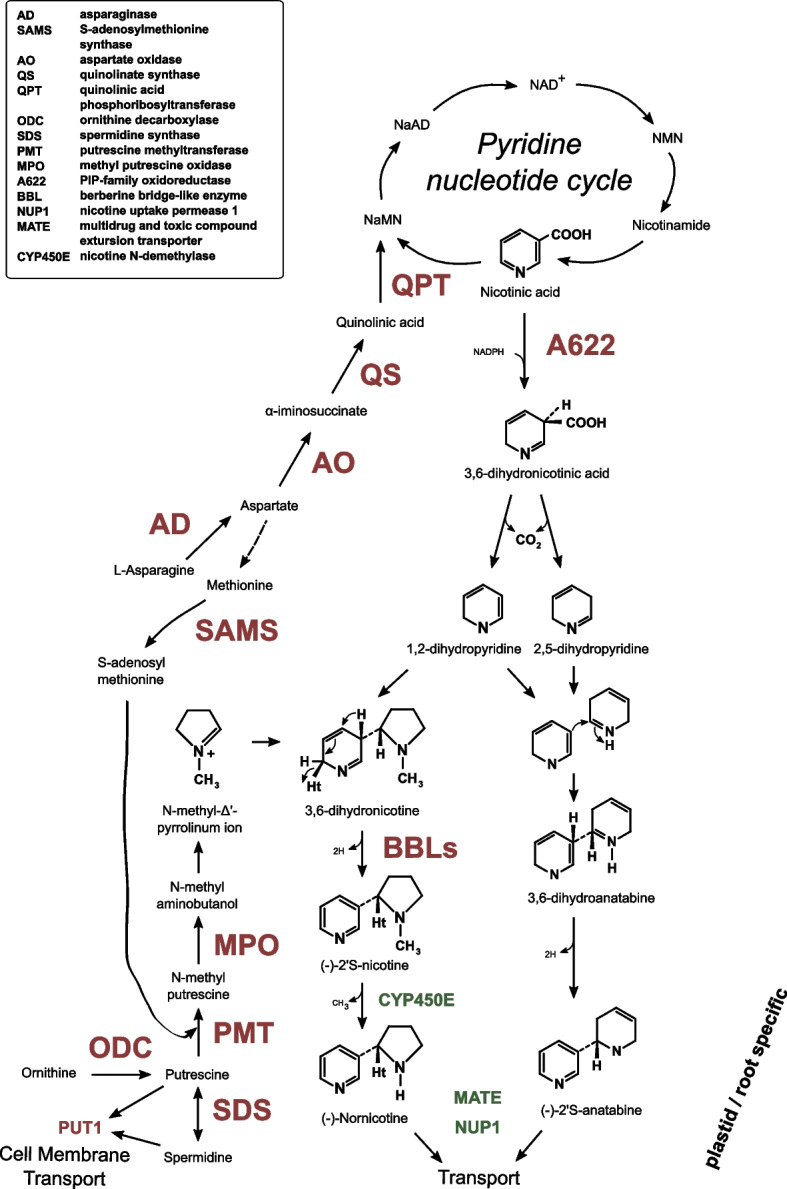
Alkaloid biosynthesis pathway breakdown. Each biochemical conversion step is represented by an arrow, with defined steps accompanied by enzyme acronyms (full enzyme names with corresponding acronyms are provided in the box). Subcellular processes taking place in plastids and are root specific are enclosed in dark red space. Enzymes that were differentially expressed after topping are colored red, while the rest are colored green. Metabolites of alkaloid biosynthesis are indicated in black, and the final biosynthesis molecules are also presented with their structural formula

The two heterocyclic rings that form nicotine originate from two amino acids: the pyridine ring from L-aspartic acid and the pyrrolidine ring from ornithine or arginine. For the early step of pyridine ring biosynthesis, L-aspartic acid is oxidized to α-iminosuccinate by aspartate oxidase (AO) that is subsequently converted to quinolinic acid by quinolate synthase (QS), which in turn forms nicotinic acid mononucleotide (NaMN) with the enzymatic action of quinolinic acid phosphoribosyltransferase (QPT), see Fig. 1 [4, 5]. NaMN enters the pyridine nucleotide cycle at this point to form nicotinic acid, thus providing a pyridine ring for nicotine biosynthesis. On the other hand, pyrrolidine ring formation starts from one of two other amino acids, arginine or non-proteinogenic ornithine. Two pathways can be followed to form the polyamine putrescine from these amino acids. Either ornithine is directly converted to putrescine by ornithine decarboxylase (ODC), see Fig. 1 [6, 7], or arginine is converted by alternative three-step arginine decarboxylase (ADC)-mediated process [8]. Putrescine is then transformed to N-methylputrescine by putrescine methyltransferase (PMT) [9, 10], which is converted further to N-methyl aminobutanol by methylputrescine oxidase (MPO) [11, 12], which is believed to cyclize spontaneously to form N-methyl-Δ’-pyrrolinium ion, a direct substrate to nicotine biosynthesis (Fig. 1). Two oxidoreductases with distinct functions are crucial in the final steps of nicotine biosynthesis. The first are isoflavone reductase-like genes (i.e., A622) that convert nicotinic acid to 3,6-dihydronicotinic acid [9, 13, 14]. At this point, it has been suggested that 3,6-dihydropyridine is decarboxylated to 1,2-dihydropyridine and 2,5-dihydropyrine, and the first reacts directly with N-methyl-Δ’-pyrrolinium ion to form nicotine [15]. However, that step requires secondary oxidoreductases—berberine bridge-like enzymes (BBLs)—that are believed to be responsible for coupling of pyridine and pyrroline rings [16], although the exact mechanism of action remains to be discovered. Finally, nicotine can be enzymatically converted to nornicotine by CYP82E4, which belongs to the cytochrome P450 protein family [17, 18].

In the case of anatabine biosynthesis, in contrast to that of nicotine, both of its rings originate from the pyridine-nucleotide. In the first step, nicotinic acid is converted to 3,6-dihydronicotinic acid by the enzymatic action of A622. With no direct evidence discovered yet and no anatabine-specific enzymes identified, previous radiolabeling and biomimetic studies point to 3,6-dihydronicotinic acid decarboxylation to 1,2-dihydropyridine and 2,5-dihydropyridine. In the subsequent step the study suggests that 3,6-dihydroanatabine can be formed from these two intermediates. In the last step of biosynthesis, the 3,6-dihydroanatabine is hydrogenated to form the final compound - anatabine [19, 20]. Based on the structure of anatabine, only the pyridine ring appears to be necessary for its biosynthesis; several studies in transgenic plants were performed to confirm this hypothesis. The first attempts to produce *N. tabacum* lines used RNA silencing approaches to suppress the enzymatic action of PMT [21–23]. Indeed, eliminating PMT translation led to suppressed pyrrolidine ring synthesis and a significant surge in anatabine accumulation. This can be explained by an overabundance of nicotinic acid-derived pyridine ring intermediates, that in absence of N-methyl-Δ’-pyrrolinium ions, have no other option than to form anatabine, the only known alkaloid that both consists of two pyridine rings and that specifically accumulates in PMT suppressed lines compared to wild-type (WT) plants. Interestingly, a build-up of anatabine was observed in untransformed tobacco BY-2 cells with a lack of nicotine [24]. It was later discovered that such cells were barely expressing N-MPO, thus leading similarly to a lack of PMT translation and suppressed pyrrolidine ring formation. This was further confirmed by generating anti-MPO lines in *N. tabacum* hairy roots, in which anatabine was produced instead of nicotine [24]. Specifically induced accumulation of primary anatabine in BY-2 cells was further confirmed when cells were treated with fungal elicitors and jasmonic acid [25]. Finally, anatabine accumulation was achieved to low levels in untransformed *N. tabacum* plants. A622 [14] and BBL [16] expression were necessary, suggesting that the final steps of alkaloid biosynthesis are similar to that of nicotine.

Finally, once alkaloids are specifically synthesized in roots [3], they need to be efficiently transported to shoots and further to leaves. Two known types of transporters are associated with this function in *N. tabacum*. Tonoplast-localized multidrug and toxic compound extrusion (MATE) family transporters were shown to be involved in vacuolar sequestration of nicotine and other alkaloids including anatabine [26]. Nicotine uptake permease 1 (NUP1), localized in plasma membranes, was also shown to be involved in specific transport by importing both nicotine and vitamin B6 into the cells [27, 28]. NUP1 was classified as nicotine-specific transporter, as demonstrated when expressed in *Schizosaccharomyces pombe* yeast cells for alkaloid uptake competition assays [27]. Still, while anatabine was supplied in 10-fold excess to nicotine, significant inhibition of NUP1 uptake was observed, suggesting that although NUP1 is primarily nicotine specific, it can also—to a lesser extent—transport other tobacco alkaloids such as anatabine [27]. Further evidence for such uptake was provided in a study of another transformed yeast *S. cerevisiae*, where NUP1 efficiently transported anatabine among other intermediates [29]. The collective evidence indicates that NUP1 is acting as an important regulatory factor of root growth and therefore is essential for overall nicotine biosynthesis potential [27, 29]. These two types of transporters may not be the only ones involved in transporting anatabine from roots to leaves in tobacco. Further studies are necessary to discover all potential genes and proteins involved in this process.

None of the naturally occurring species accumulate anatabine as their major alkaloid [30]. It was postulated that this is due to its relative lower toxicity to insects compared to the other alkaloids, which makes anatabine evolutionary unfavorable as the predominant toxic agent against predators compared to nicotine. This was demonstrated in wild *Nicotiana* species, *N. sylvestris* and *N. attenunata*, where suppression of PMT activity and higher anatabine levels instead of nicotine resulted in greater susceptibility to herbivore attack [31, 32]. A hypothesis was proposed that since anatabine cannot be regarded as an effective defense molecule against insects, it must fulfill another function, possibly as a mechanism to lower cell toxicity in a situation of unbalanced pyrrolinium ring biosynthesis. This would prevent overaccumulation of nicotinic acid that is known to be toxic in *Nicotiana* [33].

In the current study, we used transgenic lines with suppressed PMT activity to identify candidate genes for anatabine biosynthesis. For the first time, full transcriptomic analyses of these lines were performed under controlled conditions and also in response to topping treatment. Topping treatment as the effective method to enhance the accumulation of alkaloids was used to further increase expression of biosynthetic genes to effectively identify differentially expressed genes. We present our results together with alkaloid quantification to reflect the metabolite changes coupled with supporting gene expression differences. Our findings indicated that MPO genes are candidates to play a significant role in anatabine production. To confirm the function of these genes and their impact on the alkaloid synthesis and transport in leaves, MPO RNA interference (RNAi) transgenic plants were generated. Several lines were produced and cultivated until the second generation (T1 plants) from which three were chosen based on the confirmed suppression of MPO gene expression. Alkaloid content was measured in mature leaves from transgenic and control plants. An almost complete drop in nicotine content was observed in all three MPO-RNAi lines, with a concurrent anatabine increase of ~ 1.6X. This current study reveals MPO as the key gene necessary for pyrroline ring formation and thus nicotine biosynthesis. In its absence, anatabine biosynthesis takes precedence, and this alkaloid is then efficiently transported and accumulated in tobacco leaves.

## Results

### UHPLC-MS alkaloid quantification

**Fig. 2:**
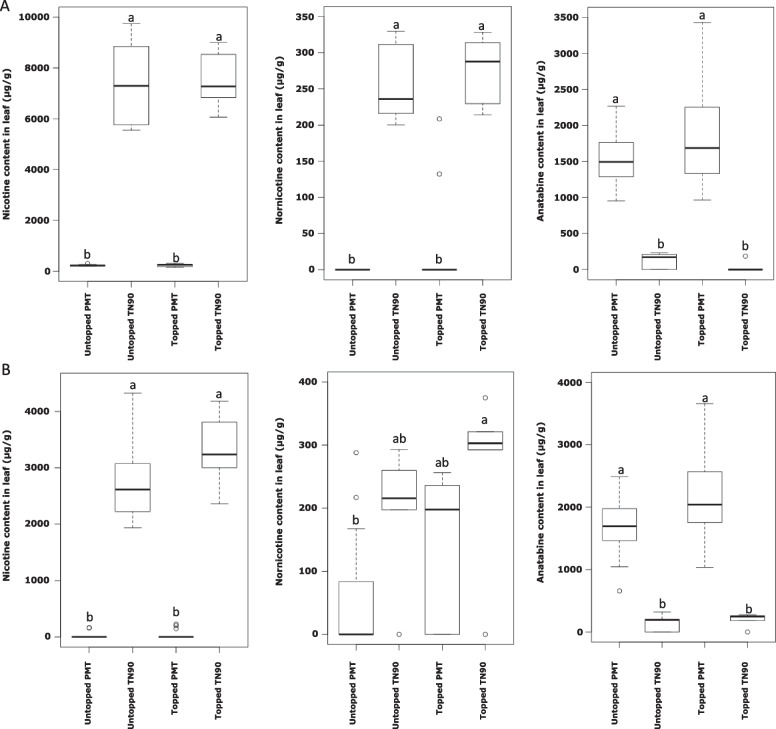
Ultra-High-Performance Liquid Chromatography coupled with Mass Spectrometry (UHPLC-MS) alkaloid quantification box-and-whisker plots. Levels of nicotine, nornicotine, and anatabine are presented in µg/g for PMT and TN90 plants and treatment (control and topped), outliers are presented as circles. Leaf and root values are presented in panels **A** and **B**, respectively. Significantly different values are designated with letters and grouped into a, b or ab (paired t-tests, ***p < 0.001)

Nicotine, nornicotine, and anatabine were quantified in the leaf and root tissues of three PMT suppressed lines (PMT1, PMT2, and PMT3) and TN90 control *N. tabacum* cultivar from untopped and topped plants, see Fig. 2. Topping had no or very low impact on leaf alkaloid chemistry in this experiment (Fig. 2A). Nicotine accumulation in all PMT suppressed lines samples represented on average only 6.69% of total alkaloids (134 ± 16 µg/g dry weight [DW]) and nornicotine only 3.01% (60 ± 14 µg/g DW), whereas anatabine was the major represented alkaloid at 90.30% (1814 ± 87 µg/g DW). The corresponding values in TN90 control were nicotine that constituted 88.85% (3077 ± 260 µg/g DW), nornicotine 6.53% (226 ± 41 µg/g DW), and only 4.9% of anatabine (169 ± 39 µg/g DW). Compared to control TN90, more substantial differences were observed in root tissue, where the three PMT suppressed lines increased their anatabine level by 30.24% on average, increasing from 1690 ± 139 (µg/g DW) in the control to 2201 ± 187 (µg/g DW) in the PMT-RNAi lines. As a reference background, the TN90 cultivar accumulated a typical proportion of alkaloids for Burley tobacco: 93.44% of nicotine (5284 ± 569 µg/g DW), 4.35% of nornicotine (246 ± 22 µg/g DW), and 2.21% of anatabine (125 ± 27 µg/g DW). The lack of change in anatabine levels in leaves from PMT suppressed lines was likely due to the alkaloid measurement being performed 24 h after topping, which was too soon to allow anatabine transfer from root to shoot.

### Gene expression analysis

Sample reads were mapped to the reference genome [34] under carefully selected criteria described in the Methods section. On average, for all leaf samples 94.56% of reads mapped to the reference genome, from which 98.33% mapped to reference gene models. The corresponding values for root samples were 82.56% and 88.64%, respectively. Sample-specific mapping statistics are provided in Supporting Table 1. There was a higher percentage of mapped reads in leaf samples compared to root samples (Supporting Table 1), which can be attributed to high expression of photosynthetic genes. The overall mapping efficiency was high and in line with other commonly used genome models [34], reflecting its high quality and accuracy, especially with the strict mapping conditions applied.

To identify candidate genes, we performed digital gene expression (DGE) in roots supported by statistical analysis (details in Methods section). To identify candidate genes, specific comparisons were made between the untopped and topped transformed PMT suppressed lines (PMT1, PMT2, PMT3), indicated by light blue cells in Table 1. The PMT RNAi lines were additionally compared with TN90 untransformed control, as indicated in Table 1 by light yellow cells. A total of 2075 genes with statistically different expression were present in at least one of these comparisons. Among the identified genes, 41.35% were assigned gene ontology (GO) biological processes, while 58.65% were of unknown function (see Supporting Table 2). When comparing PMT1, PMT2, and PMT3 lines with TN90 control, the tendencies of the number of differentially expressed genes were: PMT-RNAi untopped versus TN90 untopped (90, 511, 122), PMT-RNAi topped versus TN90 topped (173, 133, 181), PMT-RNAi untopped versus TN90 topped (291, 214, 527), and PMT-RNAi topped versus TN90 untopped (724, 911, 279), with the latter two having the highest number, see yellow cells in Table 1. The highest number of differentially expressed genes was observed when comparing PMT-RNAi lines to TN90 control plants with different topping treatments. It is also notable that when comparing untopped to topped plants, PMT-RNAi lines showed higher numbers of differentially expressed genes (229, 192, 343) than TN90 control (34), see blue cells in Table 1. It is also worth noticing that the average gene expression differences between PMT-RNAi lines were smaller than when comparing them with TN90 control. This is reflected by the number of differentially expressed genes between PMT-RNAi lines to each other (red and blue cells, average of 217 genes), which were far smaller than PMT-RNAi lines to TN90 (yellow cells, average of 346 genes). Some of the variation can be explained due to nature of plant gene expression, where noise is widespread even in the absence of environmental and genetic variation [35]. However, in the case of gene expression analysis, it is magnitude that makes the difference: comparison of PMT-RNAi lines to TN90 yielded on average 1.59X more genes than comparisons of PMT-RNAi lines to each other. The additional 0.59X variation was observed over what it would be when comparing PMT-RNAi lines of same genotype. It is partly due to alkaloid biosynthesis genes (PMT, spermidine synthase [SDS]), whereas the rest consists of genes with general or unknown functions. These may very well be tightly correlated with specific metabolic changes in transformed lines; however, elucidating their roles will require more gene-specific studies.

**Table 1:**
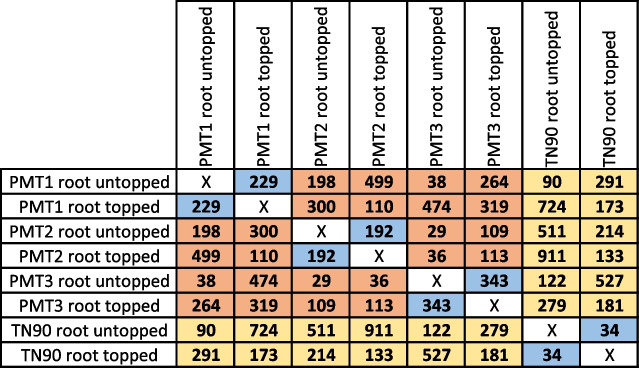
Summary of pairwise comparison by empirical analysis of digital gene expression (DGE) in root samples. Numbers of differentially expressed genes between PMT lines (PMT1, PMT2 and PMT3) untopped vs. topped treatments are represented in blue cells, while between PMT lines (PMT1, PMT2, and PMT3) vs. TN90 in both untopped and topped conditions are shown in yellow cells. Red cells correspond to comparisons between PMT lines and were not used for downstream analyses. TN90 untopped vs. topped comparison results are shown in green cells

Among the 2075 differentially expressed genes identified (listed in Supporting Table 2), 35 could be potentially associated with known alkaloid biosynthesis metabolism. These genes, their chromosome number, gene length, and RPKM expression values are listed in Table 2. Each gene was also annotated with Swiss-Prot or Solyc identifiers as described in the Methods section. Candidate genes (asparaginase [AD], aspartate oxidase [AO], QS, QPT, ODC, SDS, PMT, A622, BBL) cover all the known enzymatic steps in pyridine and pyrrolidine ring formation, as well as the final steps of nicotine biosynthesis, as shown in Fig. 1.

**Table 2:**
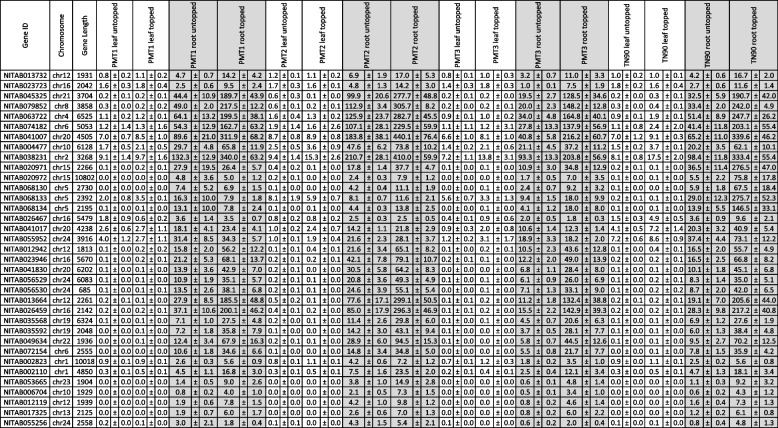
List of candidate genes identified by statistical comparison with empirical analysis of digital gene expression (DGE) in roots. Gene ID, chromosome number, gene length together with RPKM values (± standard error) for PMT lines (PMT1, PMT2, PMT3) and TN90 are presented for root and leaf tissues, as well as for untopped and topped plants. For each gene, Swiss-Prot and best corresponding tomato Solyc identifiers are provided together with reference numbers in Supporting Table 2

When comparing gene expression in roots of untopped and topped plants, for pyrrolidine ring formation the major difference affecting nicotine biosynthesis was the induced downregulation of five PMTs (NITAB068130, NITAB068133, NITAB068134, NITAB020971, and NITAB020972), in line with the expected suppressed function in PMT-RNAi lines compared to TN90 (see Table 2). Genes upregulated in topped versus untopped plants appeared exclusively in TN90: three SDS genes (NITAB026467, NITAB041017, and NITAB055952), two genes annotated as ODC (NITAB012942 and NITAB023946), and three as amine oxidase (probable MPO; NITAB041830, NITAB056529, and NITAB056530, see below). Upregulation of genes involved in pyridine ring formation was also observed in PMT-RNAi lines and TN90: two AD genes (NITAB013732 and NITAB023723) encoding enzymes that possibly convert L-asparagine to aspartate as a first step, two AO genes (NITAB045325 and NITAB079852) for the conversion of aspartate to alpha-iminosuccinate, two QS genes (NITAB063722 and NITAB074182), and one QPT gene (NITAB041007). Indirectly affecting this part of the alkaloid biosynthesis, we also observed upregulation of two S-adenosylmethionine synthases genes (SAMS; NITAB004477 and NITAB038231) that directly supply the co-factor S-adenosylmethionine necessary for the PMT enzymatic reaction, among other methyltransferases [36]. Additional transcripts were detected in root samples of topped plants, likely playing a role in the final alkaloid biosynthesis steps: two A622 genes (NITAB013664 and NITAB026459) and four BBLs (NITAB035568, NITAB035592, NITAB049634, and NITAB072154). One transport candidate was also upregulated, the polyamine transporter PUT1 (NITAB002823). Finally, several genes belonging to the cytochrome P450 family were upregulated in root samples in PMT-RNAi lines and TN90 control in response to topping (NITAB002110, NITAB053665, NITAB006704, NITAB012119, NITAB17325, and NITAB055256).

**Fig. 3:**
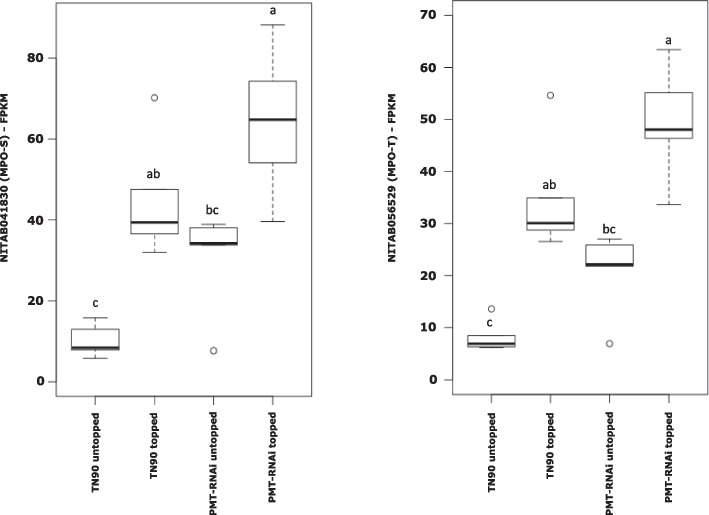
NITAB041830 (MPO-S) and NITAB056529 (MPO-T) box-and-whisker plots of the expression in N. tabacum PMT-RNAi suppressed lines. The data units are in FPKM resulting from RNAseq analyses of roots from greenhouse topped plants. Significantly different values are designated with letters and grouped into a, ab, bc or c (paired t-tests, ***p < 0.001), outliers are presented as circles

Comparison of gene expression in roots revealed that NITAB041830 and NITAB056529 transcripts corresponding to NtMPO-S and NtMPO-T genes, respectively, were upregulated in PMT-RNAi suppressed lines, in both topped and untopped plants (see Table 2, Fig. 3). NITAB041830 and NITAB056529 are primary expressed in the root tissue (Table 2). These genes were selected for further analysis to ascertain the effects of their suppression on tobacco leaf alkaloid phenotype.

### RNAi suppression of MPO genes

**Fig. 4:**
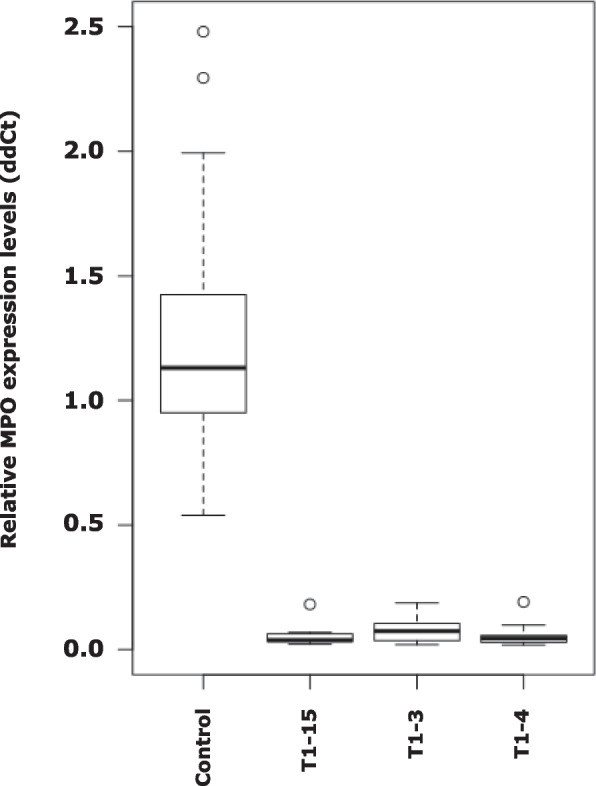
Box-and-whisker plots of the relative expression of MPO in control and RNAi lines. Lines T1-3, T1-15, and T1-4 were selected, based on the very low expression of MPO genes NITAB041830 (MPO-S) and NITAB056529 (MPO-T), outliers are presented as circles

To investigate the function of MPO genes within the alkaloid pathway and by extension the synthesis of anatabine and its subsequent transport and accumulation in leaves, MPO-RNAi plants were generated using specifically designed inserts as RNAi-constructs. Several lines were produced and cultivated until the second generation (T1 plants), see Fig. 4. Based on the gene expression levels, three MPO-T1 lines (-3, -4 and -15) were selected for subsequent analyses. No significant changes were observed in physiology and biomass between chosen transformed and control plants.

**Fig. 5:**
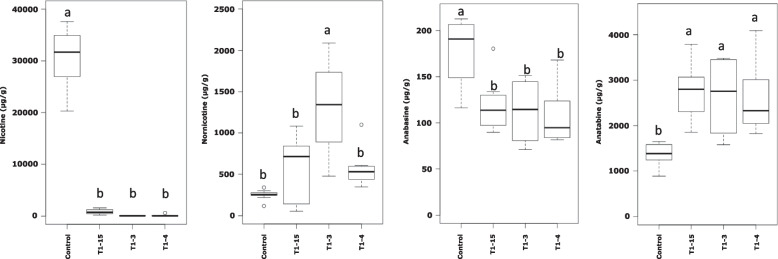
Box-and-whisker plots of the alkaloids measured by Ultra-High-Performance Liquid Chromatography coupled with Mass Spectrometry (UHPLC-MS, lower panels), from 8 WT plants, 3 independent MPO-RNAi lines, 10 plants each. Significantly different values are designated with letters and grouped into a, or b (paired t-tests, ***p < 0.001), outliers are presented as circles

We measured the nicotine and anatabine contents in the mature plant leaves (lamina) of control (WT) plants and the three MPO-RNAi suppressed lines. An almost complete drop in nicotine content was observed in all MPO-RNAi lines, with a concomitant increase of anatabine content (~1.6X), see Fig. 5. The qualitative impact on anatabine accumulation in leaves was comparable in PMT and MPO suppressed plants.

## Discussion

As reported in previous studies comparing control plants and transgenic lines with suppressed PMT activity [21–23], we also observed an alkaloid content shift from nicotine (3077 ± 260 µg/g to 134 ± 16 µg/g DW) to anatabine (169 ± 39 µg/g to 1814 ± 87 µg/g DW). Interestingly, there have been no topping studies using such transgenic lines followed by alkaloid and transcriptomic analyses. In fact, a 2020 report was the first to analyze global gene expression in response to topping. The authors observed upregulation of all known alkaloid biosynthetic genes, with the exception of QPT. However, QPT was significantly upregulated in our experiments. The current study focused on the early elicitation response (24 h after treatment) at both the alkaloid and transcriptomic levels, to identify the early response elements to topping in PMT suppressed lines and TN90 control. Indeed, such treatment was shown to induce anatabine accumulation in leaves of PMT suppressed lines [22]. As we observed, topping was more efficient in promoting alkaloid accumulation at the root level.

Differential analysis of gene expression revealed two major tendencies as summarized in Table 1. Primarily, the number of significantly different expressed genes in roots was higher than any other combination when comparing untopped versus topped treatments, thus reflecting the early response also observed at the metabolite level. Secondly, the comparison of PMT lines with TN90 control indicated that the primary significant differences in Table 2 were PMT genes, as expected. However, it is also interesting that similar differences were observed after topping treatment was applied. In fact, most of the genes listed in Table 2 were significantly differentially expressed only after topping treatment. This suggests that to observe differences related to the mechanism of alkaloid accumulation, studies should include damaged plants and not just *Nicotiana* plants growing under optimal conditions. Indeed, this is certainly a reason why we discovered transcripts that previous studies did not report [37]. AD and MPO genes were highly expressed in response to topping of PMT transgenic lines. Considering *AD*s, we can posit a possible explanation since they are involved in the early stage of pyridine ring biosynthesis. The case for MPOs is more puzzling, as these enzymes are directly involved in pyrrolidine ring formation. However, as MPO activities occur immediately after PMTs within the pyridine pathway, the results may suggest that their regulation is directly dependent on topping treatment and subsequent hormonal responses rather than on N-methylputrescine metabolite availability.

Significantly lower SDS levels were observed in transgenic lines compared with control. This could be attributed to lower putrescine availability in control plants. We can hypothesize that putrescine is plentiful in PMT lines, as it is not a substrate for the PMT reaction, so less SDS conversion of spermidine to putrescine is necessary. An appropriate balance of spermidine and spermine is essential for undisturbed plant growth and development [38], so relatively more SDS activity is required as compared to transgenic lines. Perturbing the polyamine balance, either by suppressing PMT activity and/or by topping, led to PUT1 transporter expression changes in roots. The PUT1 transporter in *Oryza sativa* was discovered as the first polyamine plastid transporter with specific preferential transport of spermidine, and as such it is essential for proper plant growth and development [39]. However, the same study showed that putrescine can be transported through the cell membrane. Upregulation of PUT1 in our experiment may be associated with an increased polyamine concentration, particularly because of increased ODC expression upon topping treatment.

Besides AD upregulated in the early stages of pyridine ring biosynthesis, we also measured the expression of other genes necessary for this process. AO, QS, and QPT genes were upregulated at the time of topping in both transgenic and control plants and were not impacted by the lack of active PMTs. This observation supports the hypothesis that AO, QS, and QPT genes are necessary for the formation of pyridine rings that serve as the backbone for both nicotine and anatabine.

Interestingly, genes responsible for final alkaloid biosynthesis were also upregulated. It was previously observed that suppression of A622 [14] and BBL [16, 40] genes leads to termination of anatabine biosynthesis, although its levels were very low at the start. In our analysis, it was observed that these two oxidoreductases play roles in PMT lines’ biosynthesis of anatabine, as reflected when looking at overexpression of A622 and BBL genes in response to topping (see Table 2). These genes are necessary, but we cannot assume that they are sufficient for final nicotine and anatabine biosynthesis. Given this gap, it is interesting to note that several genes from the cytochrome P450 family were differentially expressed in our study, including two from the CYP71D subfamily. Notably, genes from this specific clade were reported as involved in alkaloid biosynthesis in previous studies, although not in tobacco itself. The function of specific cytochrome P450s (11 discovered to date) in Madagascar periwinkle (*Catharanthus roseus*) was proven to be essential for monoterpene indole alkaloid (MIA) biosynthesis, among them vinblastine and vincristine [41]. There are genes classified as CYP71D subfamily members in MIA biosynthesis, such as tabersonine 16-hydroxylase (two isoforms: CYP71D12 and CYP71D351) [42–44] and 16-methoxytabersonine 3-oxygenase (CYP71D1) [45, 46] were discovered. It is therefore possible to speculate that candidates in our study belonging to the same CYP71D7 subfamily may also be involved in alkaloid biosynthesis. Confirming this hypothesis would require further transformation and protein activity studies. Finally, three additional candidate genes were upregulated in roots in response to topping and were annotated as homologs of *Arabidopsis thaliana* cytochrome CYP94C1 (see Table 2). These cytochrome P450 genes were shown to catalyze the oxidation step of jasmonoyl-isoleucine (JA-Ile) to form the carboxy-derivative 12COOH-JA-Ile, reflecting catalytic turnover of the hormone [47].

We investigated the action of MPOs in anatabine biosynthesis in detail. Previous studies showed that *MPO* suppression by creating transgenic lines with RNA-silenced MPOs in *N. tabacum* hairy roots hindered nicotine synthesis and promoted anatabine accumulation [24]. It has not yet been shown how fully grown plants with suppressed MPO will act and which alkaloids would accumulate in roots and leaves. For the first time, we demonstrated that plants with constitutively suppressed MPOs almost completely reduce nicotine synthesis but also redirect the process towards anatabine production. Previous studies reported that both MATE and NUP1-type transporters show affinity towards nicotine and can serve as effective transport proteins [26–29]. They most probably act in MPO suppressed lines and are expressed, yet more detailed transport focused studies are necessary to confirm their affinity towards anatabine. Furthermore, several questions concerning anatabine biosynthesis remain to be answered, particularly the specific actions of BBL genes and possibly other unidentified ones that are necessary for the process. Future studies should also focus on the mode of anatabine transport, which clearly impacts anatabine accumulation in leaves.

## Conclusions

The final steps of nicotine and nornicotine biosynthesis are essentially governed by the same mechanism, as revealed by gene expression analysis of transgenic lines with suppressed PMT activity. Nornicotine is a demethylated form of nicotine. Genes involved in both pyridine ring biosynthesis and final alkaloid formation with help of two oxidoreductases were upregulated to a similar extent in both PMT lines and control TN90 cultivar. What appeared to be different between transgenic and control plants, besides PMT activity, was specific regulation of the polyamine balance as observed with differential SDS expression. Interestingly, MPO expression was significantly higher in all plants subjected to topping treatment. Furthermore, upon topping upregulation not related to the lack of active PMT of two asparaginases and cytochrome P450 genes were identified in the transcriptome analyzed, suggesting entirely new possibilities to regulate *N. tabacum* alkaloid content. In the final part of our study, we produced transgenic lines with suppressed MPO expression. It resulted in an almost complete halt in nicotine accumulation while anatabine content was increased by ~1.6X. This is the first concrete proof that suppression of MPO alters the alkaloid pool in favor of anatabine and that this anatabine is efficiently transported from roots to tobacco leaves. Such transport was previously suggested and tested for specific transporters as MATE and NUP1 [26–29]. It is most probably the case here, where TN90 burley tobacco served as the parental plant for transformed lines, but we cannot say that this would be replicable for other tobacco varieties or species. Many plants from the *Nicotiana* genus accumulate high amounts of alkaloids in roots, yet that does not translate to effective transport into leaves [30]. Further studies where specific transporter genes are sequenced and suppressed/knocked-out or further expressed would provide a solid base to understand anatabine transport in tobacco. It would also be beneficial to measure in the future studies other compounds to reveal a more complete picture of biosynthesis, such as polyamines, amino acids and other intermediates in alkaloid biosynthesis.

## Methods

### Plant cultivation

Seeds of *N. tabacum* TN90 cultivar were taken from internal collection. TN90 seeds are also publically available at US Nicotiana Germplasm Collection (designated as PI 543792, Nicotiana tabacum L., 'TN 90') and are freely available for use and purchase for professional plant breeders and other career research scientists (https://www.ars-grin.gov/collections). Transgenic TN90 lines PMT1 (06TN2046), PMT2 (06TN2048) and PMT3 (06TN2052) were obtained from Altria Client Services LLC (Richmond, VA, USA). PMT lines were produced using Agrobacterium-mediated transformation under the control of 35S as the constitutive promotor, as described in the patent WO2015157359A1 [48]. Prior to germination, seeds were sterilized with the vapor chlorine gas method; 50 mL of 5% final chlorine was placed together with seed glass tubes in a bell jar. Subsequently, 3 mL of hydrochloric acid (37%) was added to the solution and seeds were incubated for 2 h. Under laminar flow hood, seeds were then placed onto growth medium in the plant growth room (24 °C, 16 h light / 20 °C, 8 h dark) for 4 weeks. The well-developed plantlets were transferred to the greenhouse and cultivated in 5-L pots in 10 replicates under an artificial light photoperiod (16-h light/8-h dark) until fully grown plants were obtained. At the time of flowering, half of TN90 and PMT plants were topped. At 24 h after topping treatment, representative, full-grown leaves and representative roots of cross diameter were collected for the analysis. All samples were immediately frozen in liquid nitrogen and preserved for metabolite and transcriptomic analyses.

### UHPLC-MS alkaloid quantification

Collected samples were lyophilized and disrupted by shaking at 400 rpm in containers with glass beads (8 h for leaves and 24 h for roots). At this point, roots that were still undisrupted were ground in the mortar to the smallest possible level. Samples for alkaloid analysis (according to the method by Kaminski et al., [30]) by ultra-high-performance liquid chromatography coupled with mass spectrometry (UHPLC-MS) were prepared by extracting approximately 25 mg of fine powder with water/methanol (3:7, with 5 mL) by agitating on a rotary shaker for 24 h, filtering (Fisherbrand™ Sterile PES Syringe Filter with pore size of 0.2 µm; Thermo Fisher Scientific, Waltham, MA, USA), and diluting 1:50 with the extraction mixture. A simultaneous determination of all six alkaloids was performed on an Vanquish Duo UHPLC system coupled to a Orbitrap IDX mass spectrometer (Thermo Fisher Scientific). Chromatographic separation was performed on an Acquity HSS T3 column (1.7 μm, 100 × 2.1 mm; Waters, Milford, MA, USA); the column temperature was set to 45 °C. The eluents were ammonium acetate in water (10 mM, pH = 8.9; eluent A) and ammonium acetate in water (10 mM; eluent B) applied as a gradient (0 min–10% B; 0.25 min–10% B; 4.25 min–98% B; 5.25 min–98% B; flow: 0.5 mL/min). The injection volume was 5 μL. Nicotine, anabasine, myosmine, nornicotine, cotinine, and anatabine eluted after 3.89, 3.27, 3.47, 2.76, 2.62, and 3.36 min, respectively, and were detected as [M + H]^+^ pseudomolecular ions after positive electrospray ionization.

### Sequencing and data analysis

With use of liquid nitrogen, tobacco samples were grinded to fine powder in mortars, and 200-mg samples were taken for RNA extraction. RNA extraction was performed by RNeasy® Plant Mini Kit (© QIAGEN N.V., Venlo, the Netherlands) for subsequent sequencing library preparation (no fragmentation) by TruSeq® Stranded Total RNA Gold (© Illumina, Inc., San Diego, CA, USA). Libraries were later sequenced with an Illumina HiSeq® 4000 System. Paired end 2 × 150 bp sequencing was performed with HiSeq® 3000/4000 PE Cluster Kits and HiSeq® 3000/4000 SBS Kits (© Illumina, Inc.).

### Gene expression analysis

The generated sequencing data were demultiplexed by Illumina BaseSpace® Clarity LIMS (© Illumina, Inc.) and subsequently imported to Qiagen CLC Genomics Workbench version 11.0.1 (CLC bio, a QIAGEN Company). Transcriptome reads were mapped to updated version the *N. tabacum* reference genome [49] using the ‘RNA-Seq Analysis’ 2.16 tool with similarity of 0.95 (S = 0.95) and fraction length of 0.95 (L = 0.95) as mapping criteria. The mismatch, insertion, and deletion costs were set to 2, 3, and 3, respectively. Global alignment was not performed, and paired distances were detected automatically. The maximum number of read hits was set to 10, and paired reads were counted as 2. RPKM values were retrieved for each gene in the reference genome, including for those without transcript models. A fusion gene table was not created. For each sample principal component analysis (PCA) was performed with the CLC ‘PCA for RNA-Seq’ tool. Gene expression analysis was performed with the ‘Empirical Analysis of DGE’ tool, where RPKM values for each gene were retrieved and compared by empirical analysis of DGE, which employs the ‘exact test’ developed by Robinson and Smyth [50] that was incorporated into the EdgeR Bioconductor package [51]. Genes with pairwise comparison p-values ≤ 0.05 and an absolute fold change  ≥ 2 were regarded as significantly different.

### RNAi transformation

The specific DNA fragment (GATCCAAATGATCCACATTATAGGAAGAATGCATTTGATGCAGGAGAAGATGGCCTTGGAAAGAATGCTCATT) selected for suppressing the expression of both copies of MPO (MPO-S and MPO-T) was cloned between the strong constitutive MMV promoter and the 3′ NOS terminator sequence of the nopaline synthase gene of *Agrobacterium tumefaciens* [52]. The burley tobacco variety TN90 was transformed using standard Agrobacterium-mediated transformation protocols [53]. Seeds were harvested from three independent T0 lines exhibiting the strongest MPO silencing. T1 plants from the 10 lines were grown in the greenhouse and selected by polymerase chain reaction (PCR) for presence of construct insertion in the genomic DNA, with the following primers (5'-3'): MMV-F (gacgtctaatcccaacttcgtc) and IPMS2-R (gacgtctaatcccaacttcgtc). To verify that the progeny displayed efficient transcript suppression, RNA was isolated from transgenic plants of each independent transformation event and their corresponding control plants, and quantitative PCR experiments were performed to assess MPO gene expression levels using the following primers (5' to 3') MPO-F1 (GTATTGATGACTTGGATCTTGTGATG), MPO-R1 (TATATTCCTTCAACTGGTCTTGCATA).

### Supplementary Information

**Additional file 1.** Sequences.

**Additional file 2:** **Supporting Table 1.** Mapping to reference genome statistics with read count as well as percentage [%] of mapped reads to genome and genes.

**Additional file 3:** **Supporting Table 2.** 2075 genes with statistically different expression were present in at least one of these comparisons.

## Data Availability

The datasets used and/or analysed during the current study are available at https://www.ncbi.nlm.nih.gov/geo/query/acc.cgi?acc=GSE229462.
