# Supplementary material for: Suppression of pyrrolidine ring biosynthesis and its effects on gene expression and subsequent accumulation of anatabine in leaves of tobacco (N. tabacum L.)
Source: BMC Genomics. 2023 Sep 4;24:516. doi: 10.1186/s12864-023-09588-8 (PMC10476381; doi:10.1186/s12864-023-09588-8)
Supplement: Supplementary file 1 — Additional file 1. Sequences. [file 12864_2023_9588_MOESM1_ESM.docx]

Sequences:

SEQ 1: NtMPO-S_CDS

ATGACAAGGGCTCAAACCTGCCATCCTTTGGACCCTTTATCTGCTGCTGAGATCTCAGTGGCTGTGGCAACTGTTAGAGCTGCCGGTGAAACACCTGAGGTCAGAGATGGGATGCGATTTATTGAGGTGGTTCTGGTAGAACCAGATAAAAGTGTAGTTGCATTGGCAGATGCATATTTCTTCCCACCTTTTCAGTCATCATTGATGCCGAGAACCAAAGGAGGATCTCAGATTCCTACTAAGCTTCCTCCAAGGAGAGCTAGGCTTATTGTTTACAATAAGAAAACAAATGAGACAAGCATTTGGATTGTTGAGCTAAACGAAGTACATGCTGCTGCTCGAGGTGGACATCACAGGGGAAAAGTCATCGCATCCAATGTTGTCCCTGATGTTCAGCCACCCATAGATGCTCAAGAGTATGCTGAATGTGAAGCTGTGGTGAAAAGTTATCCTCCCTTTCGAGACGCAATGAGGAGAAGGGGTATTGATGACTTGGATCTTGTGATGGTTGACCCTTGGTGTGTTGGTTATCATAGTGAGGCTGATGCTCCTAGCCGCAGGCTCGCGAAACCACTTGTATTCTGCAGGACAGAGAGTGACTGCCCAATGGAAAATGGATATGCAAGACCAGTTGAAGGAATATATGTGCTTGTTGATGTACAAAACATGAAGATTATAGAATTTGAAGACCGAAAACTTGTACCATTACCTCCAGTTGACCCACTGAGGAACTACACTGCTGGTGAGACAAGAGGAGGGGTTGATCGAAGTGATGTGAAACCCCTACATATTATTCAGCCTGAGGGTCCAAGCTTTCGTATCAGTGGAAACTACGTAGAGTGGCAGAAGTGGAACTTTCGGATTGGTTTCACCCCTAGAGAGGGTTTAGTTATACACTCTGTGGCGTATCTTGATGGTAGCAGAGGTCGTAGACCAATAGCACATAGGTTGAGTTTTGTAGAGATGGTTGTCCCCTATGGAGATCCAAATGATCCACATTATAGGAAGAATGCATTTGATGCAGGAGAAGATGGCCTTGGAAAGAATGCTCATTCACTGAAGAGGGGATGTGATTGTTTAGGGTACATAAAGTACTTTGATGCCCATTTCACAAACTTTACCGGAGGAGTTGAAACGACTGAAAATTGTGTATGCTTGCATGAAGAAGATCACGGAATGCTTTGGAAGCATCAAGATTGGAGAACTGGCCTTGCTGAAGTTAGACGGTCTAGGCGACTAACAGTGTCTTTTGTTTGTACAGTGGCCAATTATGAATATGCATTCTACTGGCATTTCTACCAGGATGGAAAAATTGAAGCGGAAGTCAAACTCACTGGAATTCTTAGTTTGGGAGCATTGCAACCTGGAGAATATCGCAAATATGGTACCACAATTTTACCAGGTTTGTATGCACCAGTTCATCAACACTTCTTTGTTGCACGAATGAATATGGCAGTTGATTGTAAGCCAGGAGAAGCACACAATCAGGTTGTTGAAGTAAATGTCAAAGTTGAAGAACCTGGCAAGGAAAATGTTCATAATAATGCATTCTATGCTGAAGAAACATTGCTTAGGTCTGAATTGCAAGCAATGCGTGATTGTGATCCATTCTCTGCTCGTCATTGGATTGTTAGGAACACAAGAACAGTAAATAGAACAGGACAGCTAACAGGGTACAAGCTGGTACCTGGTCCAAACTGTTTGCCACTGGCTGGTCCTGAGGCGAAATTTTTGAGAAGAGCTGCATTTCTGAAGCACAATCTATGGGTTACACAATATGCACCTGGAGAAGATTTTCCAGGAGGAGAGTTCCCTAATCAAAATCCCCGTGTTGGCGAGGGATTAGCTTCTTGGGTCAAGCAAGACCGGCCTCTGGAAGAAAGTGATATTGTTCTCTGGTATATTTTTGGAATCACACATGTTCCTCGGTTGGAAGACTGGCCTGTTATGCCAGTAGAACACATTGGTTTTGTGCTACAGCCACATGGATACTTTAACTGCTCTCCGGCTGTTGATGTCCCTCCGCCCTTTGCATGCGACTCAGAAAGCAGAGACAGTGATGTTACTGAAACTAGTGTAGCAAAGTCCACTGCCACTAGCTTGCTGGCCAAGCTTTGAATGTTTCGTTTATCCTAACATGAGTCCTCCTCGATCGCCTATTTACCTACGGATACCAAACTTCATTTTTCTTTTGATAGAGTATTGAATTAGTTGGTTCAGGAACATGGTTTTGACTAGTCGCATATATGGCACGTTTAAGCAAAGCAAGTCCCTTTGTGTATTGATCTTGAATAAAGCATGTTATAGGGAAAAACTCATAAATGTCGATCTTTGGTAACTCTCGGTCTTTGCATTTCATTTTGTACTATAGTCTATTTCACAAATCCTGGCTATTTGTACTGTGTTCCAA

SEQ 2: NtMPO-S_PROTEIN

MTRAQTCHPLDPLSAAEISVAVATVRAAGETPEVRDGMRFIEVVLVEPDKSVVALADAYFFPPFQSSLMPRTKGGSQIPTKLPPRRARLIVYNKKTNETSIWIVELNEVHAAARGGHHRGKVIASNVVPDVQPPIDAQEYAECEAVVKSYPPFRDAMRRRGIDDLDLVMVDPWCVGYHSEADAPSRRLAKPLVFCRTESDCPMENGYARPVEGIYVLVDVQNMKIIEFEDRKLVPLPPVDPLRNYTAGETRGGVDRSDVKPLHIIQPEGPSFRISGNYVEWQKWNFRIGFTPREGLVIHSVAYLDGSRGRRPIAHRLSFVEMVVPYGDPNDPHYRKNAFDAGEDGLGKNAHSLKRGCDCLGYIKYFDAHFTNFTGGVETTENCVCLHEEDHGMLWKHQDWRTGLAEVRRSRRLTVSFVCTVANYEYAFYWHFYQDGKIEAEVKLTGILSLGALQPGEYRKYGTTILPGLYAPVHQHFFVARMNMAVDCKPGEAHNQVVEVNVKVEEPGKENVHNNAFYAEETLLRSELQAMRDCDPFSARHWIVRNTRTVNRTGQLTGYKLVPGPNCLPLAGPEAKFLRRAAFLKHNLWVTQYAPGEDFPGGEFPNQNPRVGEGLASWVKQDRPLEESDIVLWYIFGITHVPRLEDWPVMPVEHIGFVLQPHGYFNCSPAVDVPPPFACDSESRDSDVTETSVAKSTATSLLAKL

SEQ 3: NtMPO-T_CDS

ATGGCCACTACTAAACAGAAAGTGACGGCACCTTCTTCTTCGACTGCTCCTTGCTGTCCTTCCACTTCCATCCTCCGTCGTGAGGCGACAGCTGCCGTTGCAGGCGTGGGCGACGGCCTGCAAAATTGGAACAACGTCCCGTCCGTGGATGATAAGCAGAAAAAGACGGCCTCATCAGCTCTAGCGTCATTGGCAAGCACTGAACCTCTTTCCTCCAATACCTCTACCAAAGGTATACAAATCATGACAAGGGCTCAGACTTGCCATCCTTTGGACCCTTTATCTGCTGCTGAGATCTCTGTGGCTGTGGCAACTGTTAGAGCTGCCGGTGAAACACCTGAGGTTAGAGATGGGATGCGATTTATTGAGGTGGTTCTGTTAGAACCAGATAAAAGTGTTGTTGCATTGGCAGATGCATATTTCTTCCCACCATTTCAGTCATCATTGATGCCCAGAACCAAAGGAGGATCTCTAATTCCTACTAAGCTTCCTCCAAGGAGAGCTAGGCTTATTGTTTACAATAAGAAAACAAATGAGACAAGCATATGGATTGTTGAGCTAAATGAAGTACATGCTGCTGCTCGAGGTGGACATCACAGGGGAAAAGTCATCTCATCCAATGTTGTCCCTGATGTTCAGCCACCCATAGATGCTCAAGAGTATGCTGAATGTGAAGCTGTGGTGAAAAGTTATCCTCCCTTTCGAGACGCAATGAGGAGAAGGGGTATTGATGACTTGGATCTTGTGATGGTTGACCCTTGGTGTGTTGGTTATCATAGTGAGGCTGATGCTCCTAGCCGCAGGCTCGCGAAACCACTTGTATTCTGCAGGACAGAGAGTGACTGCCCAATGGAAAATGGATATGCAAGACCAGTTGAAGGAATATATGTGCTTGTTGATGTACAAAACATGCAGATTATAGAATTTGAAGACCGAAAACTTGTACCGTTACCTCCAGCTGATCCACTGAGGAACTACACTGCTGGTGAGACAAGAGGAGGGGTTGATCGAAGTGATGTGAAACCCCTACATATTATTCAGCCCGAGGGTCCAAGCTTTCGTATCAGTGGAAACTACATAGAGTGGCAGAAGTGGAACTTTCGGATTGGTTTCACCCCTAGAGAGGGTTTAGTTATACACTCTGTGGCGTATCTTGATGGTAGCAGAGGTCGCAGACCAATAGCACATAGGTTGAGTTTTGTAGAGATGGTTGTCCCTTATGGGGATCCAAATGATCCACATTATAGGAAGAATGCATTTGATGCAGGAGAAGATGGCCTTGGAAAGAATGCTCATTCACTGAAGAGGGGATGTGATTGTTTAGGGTACATAAAGTACTTTGATGCCCATTTCACAAACTTTACGGGAGGAGTTGAAACGACTGAAAATTGTGTATGCTTGCATGAAGAAGATCACGGAATGCTTTGGAAGCATCAAGATTGGAGAACTGGCCTTGCTGAAGTTAGACGGTCTAGGCGACTAACGGTGTCTTTTGTTTGTACAGTGGCCAATTATGAATATGCATTCTACTGGCACTTCTACCAAGATGGAAAAATTGAAGCGGAAGTCAAACTCACTGGAATACTTAGTTTGGGAGCATTGCAACCTGGAGAATATCGCAAATATGGTACCACAATTTTACCAGGGTTGTATGCACCAGTTCATCAACACTTCTTTGTTGCGCGAATGAATATGGCAGTTGATTGTAAGCCAGGAGAAGCACACAATCAGGTTGTTGAAGTAAATGTCAAAGTTGAAGAACCTGGCAAGGAAAATGTTCACAATAATGCATTCTATGCTGAAGAAACATTGCTTAGGTCTGAATTGCAAGCAATGCGTGACTGTGATCCATTCTCTGCTCGTCATTGGATTGTTAGGAACACAAGAACTGTAAATAGAACAGGACAGCTAACAGGGTACAAGCTGGTACCTGGTCCAAACTGTTTGCCATTGGCTGGTCCTGAGGCGAAATTTTTGAGAAGAGCTGCATTTCTGAAGCACAATCTATGGGTTACACAATATGCACCTGGAGAAGAATTTCCAGGAGGAGAGTTCCCTAATCAAAATCCCCGTGTTGGCGAGGGATTAGCTTCATGGGTCAAGCAAGACCGGCCTCTAGAAGAAAGTGATATTGTTCTCTGGTATATTTTTGGAATCACACATGTTCCTCGGTTGGAAGACTGGCCTGTTATGCCAGTGGAACACATTGGTTTTGTGCTACAGCCACATGGATTCTTTAACTGCTCTCCGGCTGTTGATGTCCCTCCGCCCTCTGCATGCGACTCGGAAAGCAGAGACAGTGATGTTACTGAAACCAGTGTAGCAAAGTCCACTGCCACTAGCTTGCTGGCCAAGCTTTGAATGGTTCTTTTATCTTCACATGAGTCCTCCTCGCCTATTTACCAACGGATAGCAAACTTCAGTTTTCTTTTGATAGAGTATTGAATTAGAACATGGTTTTGACTAGACTCATATATGGCACGTTTAAGCAAAGCAAGTCCCTTT

SEQ 4: NtMPO-T_PROTEIN

MATTKQKVTAPSSSTAPCCPSTSILRREATAAVAGVGDGLQNWNNVPSVDDKQKKTASSALASLASTEPLSSNTSTKGIQIMTRAQTCHPLDPLSAAEISVAVATVRAAGETPEVRDGMRFIEVVLLEPDKSVVALADAYFFPPFQSSLMPRTKGGSLIPTKLPPRRARLIVYNKKTNETSIWIVELNEVHAAARGGHHRGKVISSNVVPDVQPPIDAQEYAECEAVVKSYPPFRDAMRRRGIDDLDLVMVDPWCVGYHSEADAPSRRLAKPLVFCRTESDCPMENGYARPVEGIYVLVDVQNMQIIEFEDRKLVPLPPADPLRNYTAGETRGGVDRSDVKPLHIIQPEGPSFRISGNYIEWQKWNFRIGFTPREGLVIHSVAYLDGSRGRRPIAHRLSFVEMVVPYGDPNDPHYRKNAFDAGEDGLGKNAHSLKRGCDCLGYIKYFDAHFTNFTGGVETTENCVCLHEEDHGMLWKHQDWRTGLAEVRRSRRLTVSFVCTVANYEYAFYWHFYQDGKIEAEVKLTGILSLGALQPGEYRKYGTTILPGLYAPVHQHFFVARMNMAVDCKPGEAHNQVVEVNVKVEEPGKENVHNNAFYAEETLLRSELQAMRDCDPFSARHWIVRNTRTVNRTGQLTGYKLVPGPNCLPLAGPEAKFLRRAAFLKHNLWVTQYAPGEEFPGGEFPNQNPRVGEGLASWVKQDRPLEESDIVLWYIFGITHVPRLEDWPVMPVEHIGFVLQPHGFFNCSPAVDVPPPSACDSESRDSDVTETSVAKSTATSLLAKL

SEQ 5: Variant MPO-S-W395*_CDS

ATGACAAGGGCTCAAACCTGCCATCCTTTGGACCCTTTATCTGCTGCTGAGATCTCAGTGGCTGTGGCAACTGTTAGAGCTGCCGGTGAAACACCTGAGGTCAGAGATGGGATGCGATTTATTGAGGTGGTTCTGGTAGAACCAGATAAAAGTGTAGTTGCATTGGCAGATGCATATTTCTTCCCACCTTTTCAGTCATCATTGATGCCGAGAACCAAAGGAGGATCTCAGATTCCTACTAAGCTTCCTCCAAGGAGAGCTAGGCTTATTGTTTACAATAAGAAAACAAATGAGACAAGCATTTGGATTGTTGAGCTAAACGAAGTACATGCTGCTGCTCGAGGTGGACATCACAGGGGAAAAGTCATCGCATCCAATGTTGTCCCTGATGTTCAGCCACCCATAGATGCTCAAGAGTATGCTGAATGTGAAGCTGTGGTGAAAAGTTATCCTCCCTTTCGAGACGCAATGAGGAGAAGGGGTATTGATGACTTGGATCTTGTGATGGTTGACCCTTGGTGTGTTGGTTATCATAGTGAGGCTGATGCTCCTAGCCGCAGGCTCGCGAAACCACTTGTATTCTGCAGGACAGAGAGTGACTGCCCAATGGAAAATGGATATGCAAGACCAGTTGAAGGAATATATGTGCTTGTTGATGTACAAAACATGAAGATTATAGAATTTGAAGACCGAAAACTTGTACCATTACCTCCAGTTGACCCACTGAGGAACTACACTGCTGGTGAGACAAGAGGAGGGGTTGATCGAAGTGATGTGAAACCCCTACATATTATTCAGCCTGAGGGTCCAAGCTTTCGTATCAGTGGAAACTACGTAGAGTGGCAGAAGTGGAACTTTCGGATTGGTTTCACCCCTAGAGAGGGTTTAGTTATACACTCTGTGGCGTATCTTGATGGTAGCAGAGGTCGTAGACCAATAGCACATAGGTTGAGTTTTGTAGAGATGGTTGTCCCCTATGGAGATCCAAATGATCCACATTATAGGAAGAATGCATTTGATGCAGGAGAAGATGGCCTTGGAAAGAATGCTCATTCACTGAAGAGGGGATGTGATTGTTTAGGGTACATAAAGTACTTTGATGCCCATTTCACAAACTTTACCGGAGGAGTTGAAACGACTGAAAATTGTGTATGCTTGCATGAAGAAGATCACGGAATGCTTTGAAAGCATCAAGATTGGAGAACTGGCCTTGCTGAAGTTAGACGGTCTAGGCGACTAACAGTGTCTTTTGTTTGTACAGTGGCCAATTATGAATATGCATTCTACTGGCATTTCTACCAGGATGGAAAAATTGAAGCGGAAGTCAAACTCACTGGAATTCTTAGTTTGGGAGCATTGCAACCTGGAGAATATCGCAAATATGGTACCACAATTTTACCAGGTTTGTATGCACCAGTTCATCAACACTTCTTTGTTGCACGAATGAATATGGCAGTTGATTGTAAGCCAGGAGAAGCACACAATCAGGTTGTTGAAGTAAATGTCAAAGTTGAAGAACCTGGCAAGGAAAATGTTCATAATAATGCATTCTATGCTGAAGAAACATTGCTTAGGTCTGAATTGCAAGCAATGCGTGATTGTGATCCATTCTCTGCTCGTCATTGGATTGTTAGGAACACAAGAACAGTAAATAGAACAGGACAGCTAACAGGGTACAAGCTGGTACCTGGTCCAAACTGTTTGCCACTGGCTGGTCCTGAGGCGAAATTTTTGAGAAGAGCTGCATTTCTGAAGCACAATCTATGGGTTACACAATATGCACCTGGAGAAGATTTTCCAGGAGGAGAGTTCCCTAATCAAAATCCCCGTGTTGGCGAGGGATTAGCTTCTTGGGTCAAGCAAGACCGGCCTCTGGAAGAAAGTGATATTGTTCTCTGGTATATTTTTGGAATCACACATGTTCCTCGGTTGGAAGACTGGCCTGTTATGCCAGTAGAACACATTGGTTTTGTGCTACAGCCACATGGATACTTTAACTGCTCTCCGGCTGTTGATGTCCCTCCGCCCTTTGCATGCGACTCAGAAAGCAGAGACAGTGATGTTACTGAAACTAGTGTAGCAAAGTCCACTGCCACTAGCTTGCTGGCCAAGCTTTGAATGTTTCGTTTATCCTAACATGAGTCCTCCTCGATCGCCTATTTACCTACGGATACCAAACTTCATTTTTCTTTTGATAGAGTATTGAATTAGTTGGTTCAGGAACATGGTTTTGACTAGTCGCATATATGGCACGTTTAAGCAAAGCAAGTCCCTTTGTGTATTGATCTTGAATAAAGCATGTTATAGGGAAAAACTCATAAATGTCGATCTTTGGTAACTCTCGGTCTTTGCATTTCATTTTGTACTATAGTCTATTTCACAAATCCTGGCTATTTGTACTGTGTTCCAA

SEQ 6: Variant MPO-S-W395*_PROTEIN

MTRAQTCHPLDPLSAAEISVAVATVRAAGETPEVRDGMRFIEVVLVEPDKSVVALADAYFFPPFQSSLMPRTKGGSQIPTKLPPRRARLIVYNKKTNETSIWIVELNEVHAAARGGHHRGKVIASNVVPDVQPPIDAQEYAECEAVVKSYPPFRDAMRRRGIDDLDLVMVDPWCVGYHSEADAPSRRLAKPLVFCRTESDCPMENGYARPVEGIYVLVDVQNMKIIEFEDRKLVPLPPVDPLRNYTAGETRGGVDRSDVKPLHIIQPEGPSFRISGNYVEWQKWNFRIGFTPREGLVIHSVAYLDGSRGRRPIAHRLSFVEMVVPYGDPNDPHYRKNAFDAGEDGLGKNAHSLKRGCDCLGYIKYFDAHFTNFTGGVETTENCVCLHEEDHGMLSTOP

SEQ 7: Variant MPO-T-W362*_CDS

ATGGCCACTACTAAACAGAAAGTGACGGCACCTTCTTCTTCGACTGCTCCTTGCTGTCCTTCCACTTCCATCCTCCGTCGTGAGGCGACAGCTGCCGTTGCAGGCGTGGGCGACGGCCTGCAAAATTGGAACAACGTCCCGTCCGTGGATGATAAGCAGAAAAAGACGGCCTCATCAGCTCTAGCGTCATTGGCAAGCACTGAACCTCTTTCCTCCAATACCTCTACCAAAGGTATACAAATCATGACAAGGGCTCAGACTTGCCATCCTTTGGACCCTTTATCTGCTGCTGAGATCTCTGTGGCTGTGGCAACTGTTAGAGCTGCCGGTGAAACACCTGAGGTTAGAGATGGGATGCGATTTATTGAGGTGGTTCTGTTAGAACCAGATAAAAGTGTTGTTGCATTGGCAGATGCATATTTCTTCCCACCATTTCAGTCATCATTGATGCCCAGAACCAAAGGAGGATCTCTAATTCCTACTAAGCTTCCTCCAAGGAGAGCTAGGCTTATTGTTTACAATAAGAAAACAAATGAGACAAGCATATGGATTGTTGAGCTAAATGAAGTACATGCTGCTGCTCGAGGTGGACATCACAGGGGAAAAGTCATCTCATCCAATGTTGTCCCTGATGTTCAGCCACCCATAGATGCTCAAGAGTATGCTGAATGTGAAGCTGTGGTGAAAAGTTATCCTCCCTTTCGAGACGCAATGAGGAGAAGGGGTATTGATGACTTGGATCTTGTGATGGTTGACCCTTGGTGTGTTGGTTATCATAGTGAGGCTGATGCTCCTAGCCGCAGGCTCGCGAAACCACTTGTATTCTGCAGGACAGAGAGTGACTGCCCAATGGAAAATGGATATGCAAGACCAGTTGAAGGAATATATGTGCTTGTTGATGTACAAAACATGCAGATTATAGAATTTGAAGACCGAAAACTTGTACCGTTACCTCCAGCTGATCCACTGAGGAACTACACTGCTGGTGAGACAAGAGGAGGGGTTGATCGAAGTGATGTGAAACCCCTACATATTATTCAGCCCGAGGGTCCAAGCTTTCGTATCAGTGGAAACTACATAGAGTGACAGAAGTGGAACTTTCGGATTGGTTTCACCCCTAGAGAGGGTTTAGTTATACACTCTGTGGCGTATCTTGATGGTAGCAGAGGTCGCAGACCAATAGCACATAGGTTGAGTTTTGTAGAGATGGTTGTCCCTTATGGGGATCCAAATGATCCACATTATAGGAAGAATGCATTTGATGCAGGAGAAGATGGCCTTGGAAAGAATGCTCATTCACTGAAGAGGGGATGTGATTGTTTAGGGTACATAAAGTACTTTGATGCCCATTTCACAAACTTTACGGGAGGAGTTGAAACGACTGAAAATTGTGTATGCTTGCATGAAGAAGATCACGGAATGCTTTGGAAGCATCAAGATTGGAGAACTGGCCTTGCTGAAGTTAGACGGTCTAGGCGACTAACGGTGTCTTTTGTTTGTACAGTGGCCAATTATGAATATGCATTCTACTGGCACTTCTACCAAGATGGAAAAATTGAAGCGGAAGTCAAACTCACTGGAATACTTAGTTTGGGAGCATTGCAACCTGGAGAATATCGCAAATATGGTACCACAATTTTACCAGGGTTGTATGCACCAGTTCATCAACACTTCTTTGTTGCGCGAATGAATATGGCAGTTGATTGTAAGCCAGGAGAAGCACACAATCAGGTTGTTGAAGTAAATGTCAAAGTTGAAGAACCTGGCAAGGAAAATGTTCACAATAATGCATTCTATGCTGAAGAAACATTGCTTAGGTCTGAATTGCAAGCAATGCGTGACTGTGATCCATTCTCTGCTCGTCATTGGATTGTTAGGAACACAAGAACTGTAAATAGAACAGGACAGCTAACAGGGTACAAGCTGGTACCTGGTCCAAACTGTTTGCCATTGGCTGGTCCTGAGGCGAAATTTTTGAGAAGAGCTGCATTTCTGAAGCACAATCTATGGGTTACACAATATGCACCTGGAGAAGAATTTCCAGGAGGAGAGTTCCCTAATCAAAATCCCCGTGTTGGCGAGGGATTAGCTTCATGGGTCAAGCAAGACCGGCCTCTAGAAGAAAGTGATATTGTTCTCTGGTATATTTTTGGAATCACACATGTTCCTCGGTTGGAAGACTGGCCTGTTATGCCAGTGGAACACATTGGTTTTGTGCTACAGCCACATGGATTCTTTAACTGCTCTCCGGCTGTTGATGTCCCTCCGCCCTCTGCATGCGACTCGGAAAGCAGAGACAGTGATGTTACTGAAACCAGTGTAGCAAAGTCCACTGCCACTAGCTTGCTGGCCAAGCTTTGAATGGTTCTTTTATCTTCACATGAGTCCTCCTCGCCTATTTACCAACGGATAGCAAACTTCAGTTTTCTTTTGATAGAGTATTGAATTAGAACATGGTTTTGACTAGACTCATATATGGCACGTTTAAGCAAAGCAAGTCCCTTT

SEQ 8: Variant MPO-T-W362*_PROTEIN

MATTKQKVTAPSSSTAPCCPSTSILRREATAAVAGVGDGLQNWNNVPSVDDKQKKTASSALASLASTEPLSSNTSTKGIQIMTRAQTCHPLDPLSAAEISVAVATVRAAGETPEVRDGMRFIEVVLLEPDKSVVALADAYFFPPFQSSLMPRTKGGSLIPTKLPPRRARLIVYNKKTNETSIWIVELNEVHAAARGGHHRGKVISSNVVPDVQPPIDAQEYAECEAVVKSYPPFRDAMRRRGIDDLDLVMVDPWCVGYHSEADAPSRRLAKPLVFCRTESDCPMENGYARPVEGIYVLVDVQNMQIIEFEDRKLVPLPPADPLRNYTAGETRGGVDRSDVKPLHIIQPEGPSFRISGNYIESTOP

SEQ 9 :AO2-S_CDS

ATGGCAACTGGTATCGCTTCAGGATGCGGACAGTTACATTTGAGGAAGCCTGTCTACTTGAGGAATAGCTATGGAAATAAAGCTCACTCTCATTCCAATGTGATTCTCAACGGCACGCAAAACCAGATCGCTTGGTCTAGTTGGGTTTCAAATGTCTTGCGAGTTAATAGAAGTAGCTATCCACAATGTCAAGTGATAAAAACAAACTGGAAGTCTCGGCGAGGAACAATCAAATCTAGCCAGCAGAGAGATGGATCAGTTACTAGGTACTTCGATTTCATTGTGATTGGTAGTGGAATTGCTGGCCTTCGATATGCACTTGAGGTTGCCAAGCATGGAACTGTGGCTGTGATAACCAAGGCTGAGCCACATGAGAGTAGCACTAACTATGCTCAAGGTGGTGTAAGTGCTGTGCTCTGCCCTTTGGATTCAGTGGAGAACCACATACAAGATACAATTGTGGCAGGTGCTTACCTCTGTGATAAGGAGACTGTTAAAGTAGTGTGTACTGAAGGACCTGAGAGAATTAGAGAACTGATCGCTATAGGTGCTTCATTCGATCATGGGGAGGACGGAAATCTGGATCTAGCCAGGGAAGGAGGCCACTCCCATCGTCGAATTGTCCATGCTGCTGATATGACTGGCAGAGAGATTGAAAGAGCCTTATTAGAGGCAGTGTTTAAGAATCCTAATATACATGTGTTTCAACACCATTTTGCTATCGATTTGTTGACCACTCAGGATGGTTCTGACGTAGTATGTCATGGCGTTGATACTATACACACGGAAACGAAGGAGGTTATAAGATTCATTTCAAAAGTGACTTTGCTAGCATCAGGTGGAGTTGGACATATCTATCCAAGTACTACTAATCCGACGGTTGCAACTGGTGATGGAATGGCTATGGCTCATCGAGCTCAAGCTGTAATTTCCAACATGGAGTTTGTGCAATTCCACCCAACTGCCTTGGCTGATGAAGGGCTTCCCAACATACCAAGTGCCAGAGAGAATGCTTTTTTGATAACTGAAGCTGTCAGAGGTGATGGAGGCATCCTTTATAACTTAGATATGGAAAGATTTATGCCAATGTATGATGAAAGAGCAGAACTTGCCCCGAGAGATGTGGTAGCAAGAAGTATAGATGACCAGCTCAAAAAGCGTGGCGAAAAGTATGTTCTTCTTGATATCAGTCACAAGCCCAGAGAGAAGGTTCTGTCCCATTTTCCTAATATAGCTGCTGAGTGTCTCCGCCATGGGTTAGACATAACACAGCAGCCGATTCCGGTGGTTCCTGCTGCTCACTACATGTGTGGTGGAGTCCGTGCTGGACTCGAGGGTGAGACTAATGTGCAAGGTCTTTATGTGGCAGGTGAAGTTGCATGTACTGGTTTACATGGTGCTAACCGACTTGCTAGCAACTCATTGCTTGAAGCACTAGTGTTTGCACGAAGAGCTGTACAGCCTTCAATTGATCATGTGAACGTGTCTAGAATTGATAACGGTGCATCAAGTTGGTGGCCGCGGCCTGTAGCCCCCCTGGCACTAGGAGATACAGTACTTAACAAAGTCATCCGTCGGACAAGGGAAGTGAGGAAAGAACTACAGTCAATCATGTGGGAATATGTTGGAATTGTTAGGTCTACCTCAAGACTAAATACTGCTGAGAAGAGAATCAAAGAGTTGGAGTTGGAATGGGAAACATACCTGTTTCAGCATGGCTGGGAACCAACAATGGTTGGAGTAGAGGCTTGTGAGATGAGGAATCTCTTCTGTTGTGCCAACCTGGTAGTTAGCAGTGCTCTTTCTCGACAAGAGAGTCGTGGGCTTCACTACACCACTGATTTTCCTCATGTTGAGGAAAGCGAGAGGTTGCCAACGGTTATCTTTCCTTCTCAGCGAAATAGCACATGGAGCACACGGCAATTACACGCGCAGCCGATAAGTTAG

SEQ 10: AO2-S_PROTEIN

MATGIASGCGQLHLRKPVYLRNSYGNKAHSHSNVILNGTQNQIAWSSWVSNVLRVNRSSYPQCQVIKTNWKSRRGTIKSSQQRDGSVTRYFDFIVIGSGIAGLRYALEVAKHGTVAVITKAEPHESSTNYAQGGVSAVLCPLDSVENHIQDTIVAGAYLCDKETVKVVCTEGPERIRELIAIGASFDHGEDGNLDLAREGGHSHRRIVHAADMTGREIERALLEAVFKNPNIHVFQHHFAIDLLTTQDGSDVVCHGVDTIHTETKEVIRFISKVTLLASGGVGHIYPSTTNPTVATGDGMAMAHRAQAVISNMEFVQFHPTALADEGLPNIPSARENAFLITEAVRGDGGILYNLDMERFMPMYDERAELAPRDVVARSIDDQLKKRGEKYVLLDISHKPREKVLSHFPNIAAECLRHGLDITQQPIPVVPAAHYMCGGVRAGLEGETNVQGLYVAGEVACTGLHGANRLASNSLLEALVFARRAVQPSIDHVNVSRIDNGASSWWPRPVAPLALGDTVLNKVIRRTREVRKELQSIMWEYVGIVRSTSRLNTAEKRIKELELEWETYLFQHGWEPTMVGVEACEMRNLFCCANLVVSSALSRQESRGLHYTTDFPHVEESERLPTVIFPSQRNSTWSTRQLHAQPIS

SEQ 11: AO2-T_CDS

ATGGCAACTGGTATCGCTTCAGGATGCGGACAGTTACACTTGAGGAAGCCTGTCTACTTGAGGAATAGCTATGGAAATAAAGCTCACTGTCATTCCAATGTGATTCTCAACGGCACGCAAAACCAGATCGCTTGGTCTAGTTGGGTTTCAAATGTCTTGCGAGTTAATAGAAGTAGCTATCCAGAATGTCAAGTGATCAAGACAAACTGGAAGTCTAGCCGAGGAACAATCAAATCGTGCCAGCAGAGAGATGGATCAGTTACTAGGTACTTCGATTTCACTGTGATTGGTAGTGGAATTGCTGGCCTTCGATATGCACTTGAGGTTGCCAAGCATGGAACTGTGGCTGTAATAACCAAGGCTGAGCCACATGAGAGTAGCACTAACTATGCTCAAGGTGGTGTAAGTGCTGTGCTCTGCCCTTTGGATTCAGTGGAGAGCCATATGCAAGATACAATTGTGGCAGGTGCTTATCTCTGTGATAAGGAGACTGTTAGAGTAGTGTGTACTGAAGGACCTGAGAGAATTAGAGAACTGATCGCTATGGGTGCTTCATTCGATCATGGGGAGGACGGAAATCTGGATCTAGCCAGGGAAGGAGGCCACTCCCATCGTCGAATTGTCCATGCTGCTGATATGACTGGCAGAGAGATAGAAAGAGCTCTATTAGAGGCAGTTTTTAAGAATCCTAATATACATGTGTTTCAACACCATTTTGCTATAGATTTGTTGACCACTCAGGATGGTTCTGACATAGTATGTCATGGCGTTGATACTATACACACGGAAACGAAGGAGGTTATAAGATTCATTTCAAAAGTGACTTTGCTGGCATCAGGTGGAGTTGGACATATCTATCCAAGTACTACTAATCCGACGGTTGCAACTGGTGATGGAATGGCTATGGCTCATCGAGCTCAAGCTGTAATTTCCAACATGGAGTTTGTGCAATTCCACCCAACTGCCTTGGCTGATGAAGGCCTTCCCAACATACCAAGTGCCAGAGAGAATGCTTTTTTGATAACTGAAGCTGTCAGAGGTGATGGAGGCATCCTTTACAACTTAGATATGGAAAGATTTATGCCAATGTATGATGAAAGAGCAGAACTTGCCCCGAGAGATGTGGTAGCAAGAAGTATAGATGACCAGCTCAAAAAGCGTGGCGAAAAGTATGTTCTTCTTGATATCAGTCACAAGCCCAGAGAGAAGGTTCTTTCTCATTTTCCTAATATAGCTGCTGAGTGTCTCCGCCATGGGTTAGACATAACACAGCAGCCGATTCCGGTGGTTCCTGCTGCTCACTACATGTGTGGTGGAGTCCGTGCTGGACTCGAGGGTGAGACTAATGTGCAAGGTCTTTATGTGGCAGGTGAAGTTGCATGTACTGGTTTACATGGTGCTAACCGACTTGCTAGCAACTCATTGCTTGAAGCACTAGTGTTTGCACGAAGAGCTGTACAGCCTTCAATTGATCATGTGAACGTGTCTAGAATTGATCACGGTGCTTCAAGTTGGTGGCCGCGGCCTGTAGCCCCCATGGTACTAGGAGATACAGTACTTAACAAAGTCATCTGTCGGACAAGGGAAGTGAGGAAAGAACTACAGTCAATCATGTGGGAATATGTTGGAATTGTTAGGTCTAACTCAAGACTAAACACTGCTGAGAAGAGAATCAGAGAGTTGGAGTTGGAATGGGAAACATACCTATTTCAGCATGGCTGGGAACCAACAATGGTTGGAGTAGAGGCTTGTGAGATGAGGAATCTCTTCTGTTGTGCCAACTTGGTAGTTAGCAGTGCTCTTTCTCGACATGAGAGTCGTGGGCTTCACTACACCACTGATTTTCCTCATGTTGAGGAAAGCGAGAGGTTGCCAACGGTCATTTTTCCTTCTCAGCGAAATAACTCATGGAGCTCACGCCAATTACACGCGCAGCCGATAAGTTAG

SEQ 12 : AO2-T_PROTEIN

MATGIASGCGQLHLRKPVYLRNSYGNKAHCHSNVILNGTQNQIAWSSWVSNVLRVNRSSYPECQVIKTNWKSSRGTIKSCQQRDGSVTRYFDFTVIGSGIAGLRYALEVAKHGTVAVITKAEPHESSTNYAQGGVSAVLCPLDSVESHMQDTIVAGAYLCDKETVRVVCTEGPERIRELIAMGASFDHGEDGNLDLAREGGHSHRRIVHAADMTGREIERALLEAVFKNPNIHVFQHHFAIDLLTTQDGSDIVCHGVDTIHTETKEVIRFISKVTLLASGGVGHIYPSTTNPTVATGDGMAMAHRAQAVISNMEFVQFHPTALADEGLPNIPSARENAFLITEAVRGDGGILYNLDMERFMPMYDERAELAPRDVVARSIDDQLKKRGEKYVLLDISHKPREKVLSHFPNIAAECLRHGLDITQQPIPVVPAAHYMCGGVRAGLEGETNVQGLYVAGEVACTGLHGANRLASNSLLEALVFARRAVQPSIDHVNVSRIDHGASSWWPRPVAPMVLGDTVLNKVICRTREVRKELQSIMWEYVGIVRSNSRLNTAEKRIRELELEWETYLFQHGWEPTMVGVEACEMRNLFCCANLVVSSALSRHESRGLHYTTDFPHVEESERLPTVIFPSQRNNSWSSRQLHAQPIS

SEQ13 : QS-S_CDS

ATGGACGCCGCAAATTTAGTCATGAAATCTTCCATGTTTTCGAAATCCCCATGTCCCGTTTTTGGTTCTAAACTCATTCCTAGAGCACCACCCTCTGTCTTTACTCTGCCTTCTACCTTTAGACCCCTCGTTAAATGCATACAAGCTTCCTTCCCACAAAACCCTGATTCCAAAATACCCTCAAACAATTCAACCTTTACGTGTTCAGCCGTGACTTCTTTCCCTTCTCAACAGTCTCAGCCTCACGCGACTTCCGATGCCAAGCTCCAACTCCTGATCTCGGAATTCCAGTCCCTCGTCGAACCAATGGACCGCGTGAAACGCCTCTTGCACTACTCCACACTCATCCCTTCAATGGATGCGTCCCTCAAAACCCCAGAGAATCGCGTGCTGGGTTGCACTACACAGGTATGGCTGCACGTGAGTTTCGATGAGGCCGAGAACAGGATGAAATTTGTGGCGGACAGTGACTCGGATATAACTAAAGGGTTTTGCGCGTGTTTGGTTTCGCTGCTGGACGGAGCTACTCCTGATGAGGTGCTGGCGTTGAAAACGGAGGACTTGAATGCTTTGAATGTTGCGGGTTTGAACGGGAAAGGATCTGCATCTAGGGCGAATACGTGGCATAACGTGTTGGTCAGCATGCAGAAAAGGACAAGGGCCTTGGTGGCGGAGCGTGAAGGCAGACCGCGCAACGAGCTCTTCCCATCTCTAGTAATCACTGCTGATGGTATCCAACCCCAAGGCAGCTACGCTGAAGCCCAGGCAAGGTTCCTGTTTCCTGATGAATCAAGAGTCCAAGAACTTGCCAGTTTGCTAAAGGAGAAGAAAATAGGAGTTGTTGCTCATTTCTACATGGACCCTGAGGTGCAAGGTGTTCTAACTGCAGCGCAGAAGCTTTGGCCCCATATACATATATCTGATTCTTTAGTCATGGCTGATAAAGCTGTCAGTATGGCAAAAGCTGGATGTGAATATATATCTGTATTGGGTGTAGATTTCATGTCAGAGAATGTGCGAGCCATTCTTGATCTAGCTGGATTCCCAGAGGTTGGAGTTTATCGGATGTCGGATGAACGCATCGGTTGCTCTTTGGCTGATGCTGCAGCCAGCCCAGCATACTTGGATTATCTTAAAACAGCTTCAACTTCTTCTCCATCTCTGCATGTTGTGTACATAAATACTTCTCTGGAGACAAAAGCATATTCTCATGAGCTTGTTCCGACTATAACATGTACTTCCTCTAATGTTGTGCAAACTATTCTGCAGGCATTTGCTGAAGTACCTGACTTGGAAGTGTTGTATGGTCCTGATACCTACATGGGTTCAAACATTGCAGAATTGTTCACCCAGATGTCCACGATGACTGATGAAGAAATTTCTGAGATACATCCTTTACACAACAGAAGCTCCATTAAATCTTTGCTTCCTCGACTCCATTATTTTCAGCATTGGTTAAGCGGAAGCTTTGAGGAAAGGACTGATCTCCTCAAAGCTTTTTTATCTGAGAACATCAATAATGCTTCGATTGCTGATGGGACATGTATTGTTCATCACCTCTTTGGTCATGAAGTTGTGGAGAACATAAATGAAATGTATGGTGATGCATTCCTTACTGCACACTTTGAAGTTCCTGGTGAAATGTTTTCCCTGGCAATGGAAGCGAAGAAAAGGGGCATGGGAGTAGTAGGTTCTACCTCGAACATACTCGATTTTATCAAAGAAAGGGTGGAAGAGGCCTTGAATAGAAACGTAGATGAACATCTTCAGTTTGTTTTAGGAACGGAATCAGGAATGATTACGGCAATAGTTGCAGCAGTCGGTAAATTACTAGGTTCTGCTGACACCTCTTCCGGTGGAGCAAAAGTAAGTGTTGAGATTGTCTTTCCTGTCTCGTCGGAATCAGTGACAAGAACATCTACCGGTTCGTCTCTGGACCAAAATAAGGTCAATATTATACCTGGAGTTGCAAGTGGAGAGGGATGTTCTCTACATGGTGGCTGTGCCTCCTGTCCATATATGAAGATGAACTCTCTTAGCTCGTTGCTAAGAGTTTGCCAGAGCTTGCCCCATGGCAAAGCCGAACTTTCAGCTTATGAGGCAGGACGATTCAGTTTGCAAACCCCCAATGGAAAACAAATTGCGGATGTTGGTTGTGAGCCGGTTCTGCACATGAGACACTTTCAGGCAACAAAGAGATTACCAGAGCAGCTAATCAATCAAATACTTCAACGATCAAGCTCCGCT

SEQ 14 : QS-S_PROTEIN

MDAANLVMKSSMFSKSPCPVFGSKLIPRAPPSVFTLPSTFRPLVKCIQASFPQNPDSKIPSNNSTFTCSAVTSFPSQQSQPHATSDAKLQLLISEFQSLVEPMDRVKRLLHYSTLIPSMDASLKTPENRVLGCTTQVWLHVSFDEAENRMKFVADSDSDITKGFCACLVSLLDGATPDEVLALKTEDLNALNVAGLNGKGSASRANTWHNVLVSMQKRTRALVAEREGRPRNELFPSLVITADGIQPQGSYAEAQARFLFPDESRVQELASLLKEKKIGVVAHFYMDPEVQGVLTAAQKLWPHIHISDSLVMADKAVSMAKAGCEYISVLGVDFMSENVRAILDLAGFPEVGVYRMSDERIGCSLADAAASPAYLDYLKTASTSSPSLHVVYINTSLETKAYSHELVPTITCTSSNVVQTILQAFAEVPDLEVLYGPDTYMGSNIAELFTQMSTMTDEEISEIHPLHNRSSIKSLLPRLHYFQHWLSGSFEERTDLLKAFLSENINNASIADGTCIVHHLFGHEVVENINEMYGDAFLTAHFEVPGEMFSLAMEAKKRGMGVVGSTSNILDFIKERVEEALNRNVDEHLQFVLGTESGMITAIVAAVGKLLGSADTSSGGAKVSVEIVFPVSSESVTRTSTGSSLDQNKVNIIPGVASGEGCSLHGGCASCPYMKMNSLSSLLRVCQSLPHGKAELSAYEAGRFSLQTPNGKQIADVGCEPVLHMRHFQATKRLPEQLINQILQRSSSA

SEQ15 : QS-T_CDS

ATGGATGCCGCAAATTTAGTCATGAAATCTTCCTTGTTTTCGAAATCCCCATGTCCCCTTTTTAGTTCTAAACTCATTCCTAGAGCACCACCCTCTGTCTTTACTCTGCCTTCTACCTTTAGACCCCTCGTTAAATGCATACAAGCTTCATTCCCACCAAACCCTGATTCCAAAAAACCCTCAAACAATTCAACCTTTACGTGTTCAGCTGTGACTTCCTTCCCTTCTCAACAATCTCAGCCTCACGCGCCTTCCGATGCCAAGCTCCAACTCCTGATCTCTGAATTCCAGTCCCTCGTCGAACCAATGGACCGCGTGAAACGCCTCTTGCACTACTCCACACTCCTCCCTCCAATGGACGCGTCCTTCAAAACCCCTGAGAATCGCGTACCGGGTTGCACTACACAGGTATGGCTGAACGTGAGTTTCGATGAGGCTGAGAACAGGATGAAATTTTTGGCGGACAGTGACTCGGAAATAACTAAAGGGTTTTGCGCGTGTTTGGTTTCGCTGCTGGACGGGGCTACTCCCGATGAGGTGCTGGCGTTGAAAACGGAGGACTTGAATGCTTTGAATGTTGCGGGGTTGAACGGGAAAGGATCGGCATCTAGGGCGAATACGTGGCATAATGTGTTGGTCAGCATGCAGAAAAGGACAAGGGCCTTAGTTGCGGAGCGTGAAGGCAGGCCGCGCGGCGAGCTCTTTCCATCTCTAGTAATCACAGCTGATGGTATCCAACCCCAAGGCAGCTACGCTGAAGCCCAGGCAAGGTTCCTGTTTCCTGATGAATCAAGGGTCCAAAAACTTGCCAATTTGCTAAAGGAGAAGAAAATAGGAGTTGTTGCTCATTTCTACATGGACCCTGAGGTGCAAGGTGTTCTAACTGCAGCGCAGAAGCTTTGGCCCCATATACATATATCTGATTCTTTAGTCATGGCTGATAAAGCTGTCAGTATGGCAAAAGCTGGATGTGAATATATATCTGTATTGGGTGTAGATTTCATGTCAGAGAATGTGCGAGCCATTCTTGATCTAGCTGGATTCCCAGAGGTTGGAGTTTATCGGATGTCGGACGAACGCATTGGTTGTTCTTTGGCTGATGCTGCAGCCAGCCCAGCATACTTGGATTATCTTAAAACAGCTTCAACTTCTTCTCCATCTCTGCATGTTGTGTACATAAATACTTCACTGGAGACAAAAGCATATTCTCATGAGCTTGTTCCGACTATAACATGTACTTCCTCTAATGTTGTGCAAACTATTCTGCAGGCATTTGCTGAAGTACCTGACTTGGAAGTGTTGTATGGTCCTGATACCTACATGGGTTCAAACATTGCGGAATTGTTCACCCAGATGTCCACGATGACTGATGAAGAAATTTCTGCGATACATCCTTTGCACAACAGAATCTCCATTAAATCTTTGCTTCCTCGACTGCATTATTTTCAGGATGGGACATGTATTGTTCATCACCTCTTTGGTCATGAAGTTGTGGAGAAGATAAATGAAATGTATGGGGATGCATTCCTTACTGCACACTTTGAAGTTCCTGGTGAAATGTTTTCCCTGGCAATGGAAGCGAAGAAAAGGGGCATGGGAGTAGTAGGTTCTACCTCGAACATACTCGACTTTATCAAAGAAAGGGTAGAAGAGTCCTTGAATAGAAACGTAGATGAACATCTTCAGTTTGTTTTGGGAACGGAATCAGGAATGATTACGGCAATAGTTGCAGCAGTCGGTAAATTACTAGGTTCTGCTGACTCCTCTTCCGGTGGAGCAAAAGTAAGTGTTGAGATTGTCTTTCCTGTCTCGTCAGAATCAGTGACAAGAACATCTACGGGTTCGCCTCTGGACCAAAATAAGGTCAATATTATACCTGGAGTTGCAAGTGGAGAGGGGTGTTCTCTACATGGTGGATGTGCCTCCTGTCCATATATGAAGATGAACTCTCTTAGCTCGTTGCTAAAAGTTTGCCAGAGCTTGCCCCATGGCAAAGCCGAACTTTCAGCTTATGAGGCAGGACGATTCAGTTTGCGAACCCCCAAGGGAAAACAAATTGCGGATGTTGGTTGTGAGCCGGTTCTGCACATGAGACACTTTCAGGCAACAAAGAGATTACCAGAGCAGCTAATCAATCAAATACTTCAACCTCGTGATAATGGACGATCAAGCTCTGCT

SEQ16 : QS-T_PROTEIN

MDAANLVMKSSLFSKSPCPLFSSKLIPRAPPSVFTLPSTFRPLVKCIQASFPPNPDSKKPSNNSTFTCSAVTSFPSQQSQPHAPSDAKLQLLISEFQSLVEPMDRVKRLLHYSTLLPPMDASFKTPENRVPGCTTQVWLNVSFDEAENRMKFLADSDSEITKGFCACLVSLLDGATPDEVLALKTEDLNALNVAGLNGKGSASRANTWHNVLVSMQKRTRALVAEREGRPRGELFPSLVITADGIQPQGSYAEAQARFLFPDESRVQKLANLLKEKKIGVVAHFYMDPEVQGVLTAAQKLWPHIHISDSLVMADKAVSMAKAGCEYISVLGVDFMSENVRAILDLAGFPEVGVYRMSDERIGCSLADAAASPAYLDYLKTASTSSPSLHVVYINTSLETKAYSHELVPTITCTSSNVVQTILQAFAEVPDLEVLYGPDTYMGSNIAELFTQMSTMTDEEISAIHPLHNRISIKSLLPRLHYFQDGTCIVHHLFGHEVVEKINEMYGDAFLTAHFEVPGEMFSLAMEAKKRGMGVVGSTSNILDFIKERVEESLNRNVDEHLQFVLGTESGMITAIVAAVGKLLGSADSSSGGAKVSVEIVFPVSSESVTRTSTGSPLDQNKVNIIPGVASGEGCSLHGGCASCPYMKMNSLSSLLKVCQSLPHGKAELSAYEAGRFSLRTPKGKQIADVGCEPVLHMRHFQATKRLPEQLINQILQPRDNGRSSSA

SEQ17 : QPT-S_CDS

ATGTTTAGAGCTATTCCTTTCACTGCTACAGTGCATCCTTATGCAATTACAGCTCCAAGGTTGGTGGTGAAAATGTCAGCAATAGCCACCAAGAATACAAGAGTGGAGTCATTAGAGGTGAAACCACCAGCACACCCAACTTATGATTTAAAGGAAGTTATGAAACTTGCACTCTCTGAAGATGCTGGGAATTTAGGAGATGTGACTTGTAAGGCGACAATTCCTCTTGATATGGAATCCGATGCTCATTTTCTAGCAAAGGAAGACGGGATCATAGCAGGAATTGCACTTGCTGAGATGATATTCGCGGAAGTTGATCCTTCATTAAAGGTGGAGTGGTATGTAAATGATGGCGATAAAGTTCATAAAGGCTTGAAATTTGGCAAAGTACAAGGAAACGCTTACAACATTGTTATAGCTGAGAGGGTTGTTCTCAATTTTATGCAAAGAATGAGTGGAATAGCTACACTAACTAAGGAAATGGCAGATGCTGCACACCCTGCTTACATCTTGGAGACTAGGAAAACTGCTCCTGGATTACGTTTGGTGGATAAATGGGCGGTATTGATCGGTGGGGGGAAGAATCACAGAATGGGCTTATTTGATATGGTAATGATAAAAGACAATCACATATCTGCTGCTGGAGGTGTCGGCAAAGCTCTAAAATCTGTGGATCAGTATTTGGAGCAAAATAAACTTCAAATAGGGGTTGAGGTTGAAACCAGGACAATTGAAGAAGTACGTGAGGTTCTAGACTATGCATCTCAAACAAAGACTTCGTTGACTAGGATAATGCTGGACAATATGGTTGTTCCATTATCTAACGGAGATATTGATGTATCCATGCTTAAGGAGGCTGTAGAATTGATCAATGGGAGGTTTGATACGGAGGCTTCAGGAAATGTTACCCTTGAAACAGTACACAAGATTGGACAAACTGGTGTTACCTACATTTCTAGTGGTGCCCTGACGCATTCCGTGAAAGCACTTGACATTTCCCTGAAGATCGATACAGAGCTCGCCCTTGAAGTTGGAAGGCGTACAAAACGAGCATGA

SEQ 18 : QPT-S_PROTEIN

MFRAIPFTATVHPYAITAPRLVVKMSAIATKNTRVESLEVKPPAHPTYDLKEVMKLALSEDAGNLGDVTCKATIPLDMESDAHFLAKEDGIIAGIALAEMIFAEVDPSLKVEWYVNDGDKVHKGLKFGKVQGNAYNIVIAERVVLNFMQRMSGIATLTKEMADAAHPAYILETRKTAPGLRLVDKWAVLIGGGKNHRMGLFDMVMIKDNHISAAGGVGKALKSVDQYLEQNKLQIGVEVETRTIEEVREVLDYASQTKTSLTRIMLDNMVVPLSNGDIDVSMLKEAVELINGRFDTEASGNVTLETVHKIGQTGVTYISSGALTHSVKALDISLKIDTELALEVGRRTKRA

SEQ 19 : QPT-T_CDS

ATGTTTAGGGCTCTTCCTTTCACTGCAACAGTGCATCCATATGCAATTACAGCTCCAAGGTTGGTGGTGAAAATGTCAGCAATAGCCACCAAGAATACAAGAGTGGAGTCATTAGAGGTGAAGCCACCAGCACACCCAACTTATGATTTAAAGGGTGTTATGCAACTTGCACTCTCTGAAGATGCTGGGAATTTAGGAGATGTGACTTGTAAGGCGACGATTCCTGTTGATATGGAATCCGATGCTCATTTTCTAGCAAAGGAAGACGGGATCATAGCAGGGATTGCACTTGCTGAGATGATATTCGCGGAAGTTGATCCTTCACTAAAGGTGGAGTGGTATGTAAATGATGGTGATAAAGTTCATAAAGGCTTGAAATTTGGCAAAGTACAAGGAAACGCTTACAACATTGTTATAGCTGAGAGGGTTGTTCTCAATTTTATGCAAAGAATGAGTGGAATAGCTACACTAACTAAGGAAATGGCAGATGCTGCACACCCTGCTTACATCTTGGAGACTAGGAAAACTGCTCCTGGATTACGTTTGGTGGATAAATGGGCGGTATTGATCGGTGGTGGGAAGAATCACAGAATGGGCTTATTTGATATGGTAATGATAAAAGACAATCACATATCTGCTGCTGGAGGTGTCGGCAAAGCTCTAAAATCTGTGGATCAGTATTTGGAGCAAAATAAACTTCAAATAGGGGTTGAGGTTGAAACCAGGACAATTGCAGAAGTACGTGAGGTTCTAGAATATGCATCTCAAACAAAGACTTCGTTGACTAGGATAATGCTGGACAATATGGTTGTTCCATTATCTAACGGAGATATTGATGTATCCATGCTTAAGGAGGCTGTAGAATTGATCAATGGGAGGTTTGATACGGAGGCTTCAGGAAATGTTACCCTTGAAACAGTACACAAGATTGGACAAACTGGTGTTACCTACATTTCTAGTGGTGCCCTGACGCATTCTGTGAAAGCACTTGACATTTCCCTGAAGATTGATACAGAGCTCGCCCTTGAAGTTGGAAGGCGTACAAAACGAGCA

SEQ 20: QPT-T_PROTEIN

MFRALPFTATVHPYAITAPRLVVKMSAIATKNTRVESLEVKPPAHPTYDLKGVMQLALSEDAGNLGDVTCKATIPVDMESDAHFLAKEDGIIAGIALAEMIFAEVDPSLKVEWYVNDGDKVHKGLKFGKVQGNAYNIVIAERVVLNFMQRMSGIATLTKEMADAAHPAYILETRKTAPGLRLVDKWAVLIGGGKNHRMGLFDMVMIKDNHISAAGGVGKALKSVDQYLEQNKLQIGVEVETRTIAEVREVLEYASQTKTSLTRIMLDNMVVPLSNGDIDVSMLKEAVELINGRFDTEASGNVTLETVHKIGQTGVTYISSGALTHSVKALDISLKIDTELALEVGRRTKRA

SEQ 21: MPO-RNAi insert:

GATCCAAATGATCCACATTATAGGAAGAATGCATTTGATGCAGGAGAAGATGGCCTTGGAAAGAATGCTCATT

SEQ 22 : Primers for qRT-PCR

MPO-F1

GTATTGATGACTTGGATCTTGTGATG

MPO-R1

TATATTCCTTCAACTGGTCTTGCATA
